# Cross-species comparison of aCGH data from mouse and human *BRCA1*- and *BRCA2*-mutated breast cancers

**DOI:** 10.1186/1471-2407-10-455

**Published:** 2010-08-24

**Authors:** Henne Holstege, Erik van Beers, Arno Velds, Xiaoling Liu, Simon A Joosse, Sjoerd Klarenbeek, Eva Schut, Ron Kerkhoven, Christiaan N Klijn, Lodewyk FA Wessels, Petra M Nederlof, Jos Jonkers

**Affiliations:** 1 Division of Molecular Biology, Netherlands Cancer Institute, Plesmanlaan 121, 1066 CX Amsterdam, The Netherlands; 2 Division of Clinical Genetics, VU Medical Center, de Boelelaan 1118, 1081 HV Amsterdam, The Netherlands; 3 Division of Experimental Therapy, Netherlands Cancer Institute, Plesmanlaan 121, 1066 CX Amsterdam, The Netherlands; 4 Skyline-Diagnostics B.V., Dr. Molewaterplein 50, 3015 GE Rotterdam, The Netherlands; 5 Central Microarray Facility, Netherlands Cancer Institute, Plesmanlaan 121, 1066 CX Amsterdam, The Netherlands; 6 Biomedical Analysis Center, Tsinghua University, Beijing, 100084, China; 7 Institute of Tumor Biology, University Medical Center Hamburg Eppendorf, Martinistrasse 52, 20246 Hamburg, Germany; 8 Faculty of Electrical Engineering, Mathematics and Computer Science, Delft University of Technology, Delft, The Netherlands

## Abstract

**Background:**

Genomic gains and losses are a result of genomic instability in many types of cancers. *BRCA1*- and *BRCA2*-mutated breast cancers are associated with increased amounts of chromosomal aberrations, presumably due their functions in genome repair. Some of these genomic aberrations may harbor genes whose absence or overexpression may give rise to cellular growth advantage. So far, it has not been easy to identify the driver genes underlying gains and losses. A powerful approach to identify these driver genes could be a cross-species comparison of array comparative genomic hybridization (aCGH) data from cognate mouse and human tumors. Orthologous regions of mouse and human tumors that are commonly gained or lost might represent essential genomic regions selected for gain or loss during tumor development.

**Methods:**

To identify genomic regions that are associated with *BRCA1*- and *BRCA2*-mutated breast cancers we compared aCGH data from 130 mouse *Brca1*^*Δ/Δ*^*;p53*^*Δ/Δ*^, *Brca2*^*Δ/Δ*^*;p53*^*Δ/Δ *^and *p53*^*Δ/Δ *^mammary tumor groups with 103 human *BRCA1-*mutated, *BRCA2-*mutated and non-hereditary breast cancers.

**Results:**

Our genome-wide cross-species analysis yielded a complete collection of loci and genes that are commonly gained or lost in mouse and human breast cancer. Principal common CNAs were the well known *MYC*-associated gain and *RB1/INTS6*-associated loss that occurred in all mouse and human tumor groups, and the *AURKA*-associated gain occurred in BRCA2-related tumors from both species. However, there were also important differences between tumor profiles of both species, such as the prominent gain on chromosome 10 in mouse *Brca2*^*Δ/Δ*^*;p53*^*Δ/Δ *^tumors and the PIK3CA associated 3q gain in human *BRCA1-*mutated tumors, which occurred in tumors from one species but not in tumors from the other species. This disparity in recurrent aberrations in mouse and human tumors might be due to differences in tumor cell type or genomic organization between both species.

**Conclusions:**

The selection of the oncogenome during mouse and human breast tumor development is markedly different, apart from the MYC gain and RB1-associated loss. These differences should be kept in mind when using mouse models for preclinical studies.

## Background

Western women have a 10-12% risk of developing breast cancer, making this disease the most common malignancy in females [1]. Approximately 5-10% of breast cancer cases can be explained by a hereditary predisposition. Between 25-40% of these cases involves the inheritance of one defective copy of either the *BRCA1 *gene or the *BRCA2 *gene [2-5] which predisposes women in these families to a ~50-80% lifetime risk of developing breast cancer and to a lesser extent ovarian cancer [1].

BRCA1 and BRCA2 proteins are implicated in DNA double strand break (DSB) repair and loss of BRCA function results in chromosomal instability, hence to cancer predisposition [6-8]. Chromosomal instability can be evaluated by array-based comparative genomic hybridization (aCGH) which measures genomic copy number alterations (CNAs) in sample (tumor) DNA relative to normal (diploid) DNA [9]. Analysis of the recurrent regions of chromosomal instability in BRCA1 or BRCA2 mutated breast tumors may point to loci and/or genes involved in development and progression of *BRCA*-associated hereditary breast cancer. To gain insight into the specific effects of BRCA1 or BRCA2 loss on genomic instability, we used aCGH profiles of genetically defined mouse models for BRCA1- and BRCA2-associated breast cancer as biological data filters to mine the human breast cancer genome [10,11]. In these mouse models breast cancer is driven by combined loss of p53 and BRCA1 or BRCA2. The mouse *p53^Δ/Δ ^*mammary tumors are a mix of basal carcinomas and carcinosarcomas, whereas the mouse *Brca1^Δ/Δ^;p53^Δ/Δ ^*and *Brca2^Δ/Δ^;p53^Δ/Δ ^*tumors are mainly basal-like. Human *BRCA2*-mutated tumors resemble sporadic tumors in that they do not skew towards any phenotype [12], whereas, 90% of human *BRCA1*-mutated tumors have a basal like phenotype [10,12,13]. Of note, tumors from our mouse models do not give rise to ER-positive luminal tumors, which is the cell type of 70-80% of human sporadic and *BRCA2-*mutated breast tumors [14,15]. It is well known that human basal-like and luminal human breast tumors have different aCGH profiles [16]; therefore, the comparison of aCGH profiles of mouse *Brca1^Δ/Δ^;p53^Δ/Δ ^*carcinomas with basal-like human *BRCA1-*mutated tumors may give results relevant for basal-like breast tumors as well as for BRCA1-loss.

Syntenic regions that are found recurrently gained or lost in both human and mouse have a greater probability of being relevant for development of BRCA-associated and/or sporadic breast cancer [17]. To analyze syntenic genomic regions for overlapping CNAs, we applied two statistical frameworks to BRCA1-deficient, BRCA2-deficient and BRCA1/2-proficient control mouse and human breast cancers: a total of six tumor groups. First we used KC-SMART [18], which identifies significantly recurrent gains and losses within each tumor group. Next, we identified syntenic CNAs that co-occurred in both the mouse and human BRCA1-mutated, BRCA2-mutated and control tumor groups. Our data indicate that amplification of the MYC locus and loss of the RB/INTS6 locus occurs in all mouse and human tumor groups, and that AURKA amplification occurs in mouse and human BRCA2-mutated tumors. We also applied *comparative*-KC-SMART which identifies gains and losses that occur significantly more often in one tumor group compared to another. We used this method to find CNAs that occur more often in BRCA1- or BRCA2-deficient breast tumors compared to BRCA-proficient control tumors. Next, we identified which of these CNAs occur both in mouse BRCA1- or BRCA2-deficient mammary tumors and in human BRCA1- or BRCA2-mutated tumors. Together, our KC-SMART and *comparative*-KC-SMART analyses highlight important genotypic similarities between mouse and human cognate cancers. However, we also find several genomic aberrations to be highly recurrent in one species but not in the other.

## Methods

### Mouse tumors

We bred cohorts of *K14Cre;Brca1*^*F/F*^;*p53*^*F/F *^female mice[10], *K14Cre;Brca2^F/F^;p53^F/F ^*female mice[11], and *K14Cre;p53*^*F/F *^female mice[10]. *Cre *recombinase expression in these mice is restricted to cytokeratin-14 (K14) expressing epithelial tissues, including mammary gland epithelium. We isolated DNA from 35 *Brca1*^*Δ/Δ*^*;p53 *^*Δ/Δ*^, 62 *K14Cre;Brca2*^*Δ/Δ*^*;p53*^*Δ/Δ *^and 33 *p53*^*Δ/Δ *^mammary tumors as well as (tumor-free) spleens from the same animals. Tumor type was scored by histopathology and by expression levels of vimentin and E-cadherin: both histopathology procedures and expression array procedures were previously described in [10]. All animal experiments were approved by the local ethical review committee.

Mouse tumor aCGH profiles were obtained using a microarray containing 3080 unique mouse bacterial artificial chromosome (BAC) clones spotted in duplicate, as described previously [19]. Hybridizations were done as described in Chung et al., 2004 with minor modifications. In brief, we labeled 2 μg of tumor DNA with Cy5 and 2 μg reference (spleen) DNA with Cy3 and vice versa for the dye swap (Universal Linkage System, Kreatech Biotechnology, Cat# EA-006) according to the manufacturer's protocol. All hybridizations were performed in a hybridization station (Tecan, Cat# Hs4800). Data, including detailed hybridization protocols were added to the public repository Array Express with accession numbers E-NCMF-34 and E-NCMF-35 [20]. The intensity of the Cy5 (tumor) signal was calculated relative to the intensity of the Cy3 (reference) signal for each spot on the array and this ratio was log2 transformed. Data was normalized by shifting these ratios by the median log2 ratio per sub array. Dye swap data (a total of four spots per data point) were combined using the Rosetta error model [21]. Each mouse tumor DNA sample was isolated from fresh tumor tissues and hybridized against a reference DNA (spleen) obtained from the same animal to avoid picking up DNA copy number variations between mice.

To compare aCGH profiles from mouse with human *BRCA1- *and *BRCA2-*mutated tumors, we used two previously published aCGH datasets [22,23]. For the cross-species comparison: we used 27 human *BRCA1-*mutated and 28 BRCA2 related breast tumors for the KC-SMART analysis, and compared them with 48 control tumors of different cell types in the *comparative-*KC-SMART analysis. Pathology, tumor type, and *BRCA1- *and *BRCA2*-mutations of human tumors are shown in Additional file 1. All experiments with human materials were approved by the local ethics review committee.

### KC-SMART and comparative-KC-SMART analysis

#### KC-SMART

(Kernel Convolution - a Statistical Method for Aberrant Region detection) determines which CNAs are significantly recurrent *within a tumor group *[18]. KC-SMART generates a Kernel Smoothed Estimate (KSE) for gains (KSE_gains_) and losses (KSE_losses_) separately across the genome and across *agroup* of tumors. Briefly, at the genomic midposition of each BAC probe, KC-SMART places a Gaussian kernel with the height of the sum of either the positive log2 values or the negative log2 values of all tumors within a tumor group. KSE curves (one for gains and one for losses) are point estimates determined by convolution of locally weighted kernel functions such that the closer the genomic distance between two probes the more they will contribute to each others convoluted values. Significance cut-offs are calculated for gains and losses separately using the distribution of KSE peak heights from randomized data (P < 0.05) as explained in [18].

#### *Comparative-*KC-SMART

detects CNAs that occur significantly more often in one tumor group *compared *to another. The algorithm creates a KSE curve for each individual tumor by placing Gaussian kernel functions with the height of the log2 value and at the genomic midposition of each probe (without separating gains and losses, as done for KC-SMART analysis). For each genomic position in the KSE curve, the KSE values from one tumor group are compared to the KSE values from another group by calculating a signal to noise ratio (SNR). We determined a cutoff that defines significant SNR values by applying a False Discovery Rate (FDR) of 0.05 on the SNR data and randomized SNR data using 6000 class-label permutations.

The width of a kernel applied to each data point determines the sensitivity of smoothing and the size of aberrations detected. To compare mouse and human tumor groups we consistently used one kernel width for all tumor profiles. We found that, when using a 20 Mb kernel width, KC-SMART best smoothed noise while detecting CNAs from aCGH profiles of both mouse and human individual tumors. To identify the genomic locations of the peaks of a KSE curve, we calculated for which position the KSE value was higher (for gains), or lower (for losses), compared to the values of its neighboring datapoints. While in many instances a peak may be a local maximum or minimum within a larger gain or loss, these local peaks are part of the data and might harbor interesting genes that drive the larger gain or loss. An R-package of the KC-SMART algorithm (which includes the *comparative-*KC-SMART algorithm) is included in Bioconductor [24].

### Combining mouse and human aCGH datasets

We used the BioMart data-mining tool in Ensembl Build 52 to cross-reference two Ensembl datasets (NCBI Build 36 and NCBI Build 37). Because NCBI Build 36 was used to map the mouse RP23-BAC clones, we mapped the genomic positions of the mouse genes using this older build. However, in order to use the most current mouse-human orthologue information, we matched the ENSMUS numbers of NCBI Build 37 with their genomic positions as listed in NCBI Build 36. We obtained a list of 19589 unique mouse-human homolog combinations. In this list we found 16679 unique human genes and 17048 unique mouse genes (one human gene may have more than one mouse homolog and vice versa). We determined which genes from this list map to the significantly gained or lost regions as determined by KC-SMART method or to the differentially gained or lost regions as determined by the *comparative*-KC-SMART method for the human and mouse tumor groups separately. Next, we queried for those genes whose homologues map to regions gained or lost in *both *the human and the mouse tumor sets. The locations of these genes in the mouse and human genome are plotted by connecting lines to their syntenic regions. Genomic locations of the overlapping syntenic regions were determined by taking the genomic position of the start of the first gene and the end of the last gene.

## Results

### 1: aCGH analysis of mouse mammary tumors

**Figure 1:**
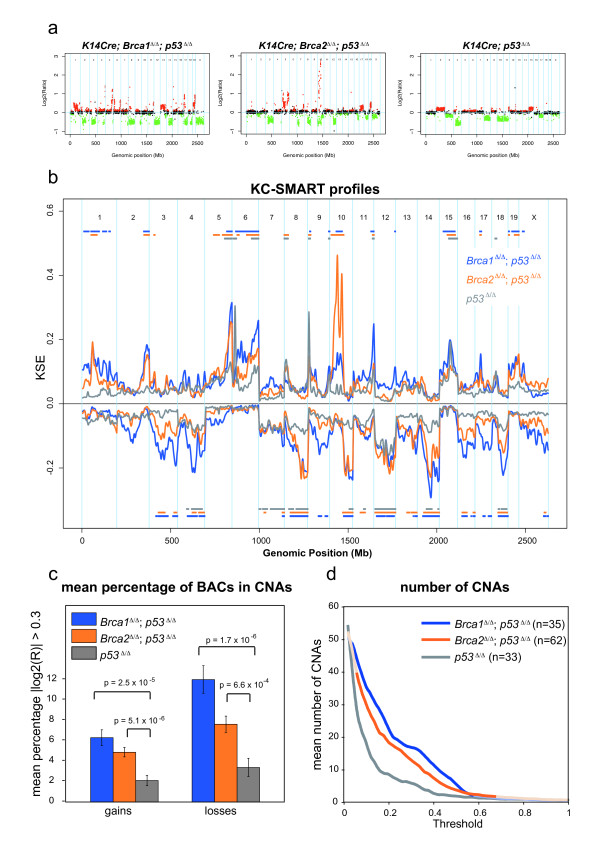
**aCGH profiles of mouse mammary tumors**. aCGH profiles typical of **(a) ***Brca1 *^*Δ/Δ*^*;p53*^*Δ/Δ*^, *Brca2*^*Δ/Δ*^*;p53*^*Δ/Δ *^and *p53*^*Δ/Δ *^mammary tumors. Each dot represents the averaged log2 ratio (y-axis) of a BAC clone plotted at its genomic position (x-axis). Red dots (gains) and green dots (losses) represent datapoints with log2 ratios significantly different from 0 as determined by the Rosetta error model [21]; black dots represent datapoints with log2 ratios not significantly different from 0. Blue vertical lines represent chromosome boundaries. **(b) **aCGH profiles from 35 *Brca1*^*Δ/Δ*^*;p53*^*Δ/Δ *^tumors (blue), 62 *Brca2 *^*Δ/Δ*^*;p53*^*Δ/Δ *^tumors (orange) and 33 *p53*^*Δ/Δ *^tumors (gray) were analyzed with KC-SMART using a kernel width of 20 Mb, for gains and losses separately. Significant CNAs are depicted in the color matched bars on top (gains) and below (losses) the Kernel Smoothed Estimate curves (KSEs) for each tumor group. **(c) **The mean percentage of BAC clones ± S.E.M. with absolute log2 ratio > 0.3 is greater across the *Brca1*^*Δ/Δ*^*;p53*^*Δ/Δ *^tumors (blue) and *Brca2 *^*Δ/Δ*^*;p53*^*Δ/Δ *^tumors (orange) tumors compared with the *p53*^*Δ/Δ *^tumors. P-values are determined by a two-tailed t-test with unequal variance. **(d) **Each individual tumor aCGH profile was smoothed using KC-SMART (kernel width: 20 Mb). For a range of thresholds, all gains exceeding a positive threshold and losses exceeding the same negative threshold were counted and averaged over each tumor group. Curves are darkened at thresholds for which the average number of CNAs per tumor group is significantly greater in the *Brca1/2*^*Δ/Δ*^*;p53*^*Δ/Δ *^tumors compared with the *p53*^*Δ/Δ *^tumors, P < 0.05 determined by a two-tailed t-test with unequal variance.

To investigate the impact of BRCA1 and BRCA2 deficiency on chromosomal instability in breast epithelial cells, we performed aCGH on mammary tumors derived from our genetically engineered mouse (GEM) models for BRCA1- and BRCA2-associated breast cancer [10,11]. Mammary tumors in these mice arose from epithelial-specific loss of p53 alone (n = 33), or in combination with BRCA1 (n = 35) or BRCA2 (n = 62). Typical examples of aCGH profiles from *Brca1*^*Δ/Δ*^*;p53*^*Δ/Δ *^, *Brca2*^*Δ/Δ*^*;p53*^*Δ/Δ *^and *p53*^*Δ/Δ *^tumors are shown in Figure 1a. All but one tumors in the *Brca1*^*Δ/Δ*^*;p53*^*Δ/Δ *^tumor group were of basal cell type, consisting of primarily high grade invasive ductal carcinoma not otherwise specified (IDC-nos; 91%), 3% carcinosarcomas and 6% adenomyoepitheliomas (previously described in [10]). Likewise, 90% of the *Brca2*^*Δ/Δ*^*;p53*^*Δ/Δ *^tumors are carcinomas and 9% are carcinosarcomas. In contrast, the *p53*^*Δ/Δ *^tumor group consisted of mixed cell types: 39% intermediate to high-grade IDC-nos, 50% carcinosarcomas and 11% adenomyoepitheliomas. Tumor type scoring was based on histopathology and E-cadherin/Vimentin expression, Table 1, (for tumor type data see Additional files 2 and 3).

**Table 1:** Tumor types according to histopathology

mouse tumor group	total scored	Carcinoma	Carcinosarcoma	Adenomyoepithelioma	NA
Brca1^Δ/Δ^;p53^Δ/Δ ^(n = 35)	34	91% (31)	3% (1)	6% (2)	1
Brca2^Δ/Δ^;p53^Δ/Δ ^(n = 62)	54	90% (49)	9% (5)	0% (0)	8
p53^Δ/Δ ^(n = 33)	28	39% (11)	50% (14)	11% (3)	5

Histopathology of *Brca1*^*Δ/Δ*^*;p53*^*Δ/Δ *^, *Brca2*^*Δ/Δ*^*;p53*^*Δ/Δ *^, *p53*^*Δ/Δ *^mammary tumors, determined by H&E and/or vimentin, SMA, as previously described in [10].

We used the KC-SMART method [18] to identify significantly recurrent genomic aberrations in the aCGH data of the mouse mammary tumor groups. Figure 1b shows Kernel Smoothed Estimate curves (KSE curves; see Methods) for gains and losses separately for the *Brca1*^*Δ/Δ*^*;p53*^*Δ/Δ*^, *Brca2*^*Δ/Δ*^*;p53 *and *p53*^*Δ/Δ *^tumor groups. Aberrations that exceeded the significance thresholds are indicated by color-matched bars above (gains) or below (losses) the KSE curves, and their genomic positions are listed in Additional file 4. Significantly recurrent gains (chromosomes 5, 6, 9-cen, 11-tel and 15) and losses (chromosomes 4, 7-tel, 8, 10-tel, 12, 14, and 18) overlapped between all tumor groups. Regional gains on chromosomes 1, 2, 10, 17, 19 and losses on chromosomes 3, 13, 16 and X were common to both *Brca1*^*Δ/Δ*^*;Trp53*^*Δ/Δ *^and *Brca2*^*Δ/Δ*^*;Trp53*^*Δ/Δ *^tumors but not to *Trp53 *^*Δ/Δ *^tumors. In addition, a high level amplicon on chromosome 10 occurred in >50% of the *Brca2*^*Δ/Δ*^*;Trp53*^*Δ/Δ *^tumors.

Compared with the *Brca1*^*Δ/Δ*^*;Trp53*^*Δ/Δ *^tumor group, the *Trp53*^*Δ/Δ *^tumor group showed more whole chromosome gains and losses, rather than regional aberrations. The *Brca2*^*Δ/Δ*^*;Trp53*^*Δ/Δ *^tumor group appeared to be an intermediate version of both extremes. To quantify the level of genomic instability for each tumor group we calculated the mean percentage of BAC clones with log2 ratio >0.3 for gains and <-0.3 for losses separately and found that this percentage was significantly higher for the *Brca1*^*Δ/Δ*^*;p53*^*Δ/Δ *^and *Brca2*^*Δ/Δ*^*;p53*^*Δ/Δ *^groups compared with the *p53*^*Δ/Δ *^group (Figure 1c). Also the *Brca1*^*Δ/Δ*^*;p53*^*Δ/Δ *^group had significantly more losses than the *Brca2*^*Δ/Δ*^*;p53*^*Δ/Δ *^group.

**Figure 2:**
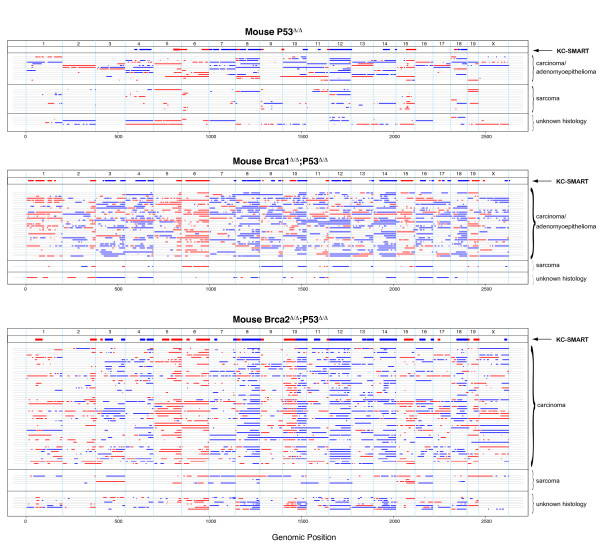
**Gains and losses of individual mouse tumors**. To visualize gains and losses of each individual mouse tumor we smoothed their individual CGH profiles with KC-SMART (without separating gains and losses) and depicted the regions which exceeded an arbitrary threshold of 0.15 in line-plots. For those tumors for which the tumor type was known, we stratified according to tumor type. For each tumor depicted in each line, the tumor type information is given in Additional file 3.

Next, to quantify the amount of CNAs in each individual tumor we smoothed their individual aCGH profiles with KC-SMART (without separating gains and losses) and determined the mean number of peaks in the KSE curves exceeding a range of KSE thresholds. We found that the average number of CNAs was significantly higher in the *Brca1*^*Δ/Δ*^*;p53*^*Δ/Δ *^tumor group compared with the *p53*^*Δ/Δ *^tumor group between thresholds of 0.04 - 0.54, and significantly higher in the *Brca2*^*Δ/Δ*^*;p53*^*Δ/Δ *^tumor group compared with the *p53*^*Δ/Δ *^tumor group between thresholds of 0.06 - 0.62 (Figure 1d). To exemplify the regions counted, we depicted the regions exceeding an arbitrary threshold of 0.15 in line-plots (Figure 2). Overall, gains and losses that occurred in the *p53*^*Δ/Δ *^tumor group also occurred in the *Brca1*^*Δ/Δ*^*;p53*^*Δ/Δ *^and *Brca2*^*Δ/Δ*^*;p53*^*Δ/Δ *^tumor groups but in a greater fraction of tumors, and with higher log2 ratios. Hence, *Brca1 *or *Brca2 *loss on top of *p53 *loss appears to aggravate the tumor profile.

**Figure 3:**
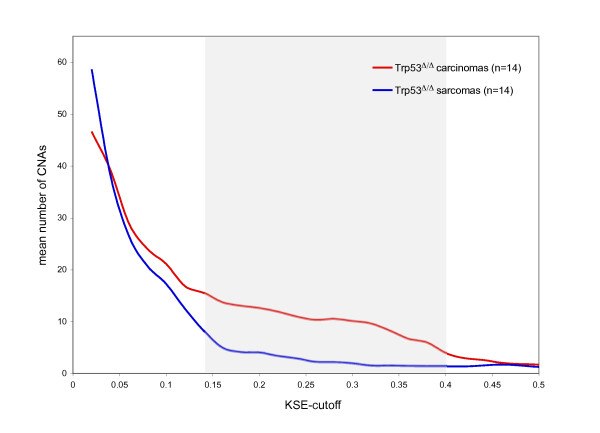
**Genomic instability of mouse p53^Δ/Δ ^mammary tumors: carcinomas and sarcomas**. The 33 *p53*^*Δ/Δ *^tumors consisted of 14 sarcomas, 14 carcinomas/adenomyoepitheliomas and the tumor type of 5 tumors was unknown. To find the difference of genomic instability between carcinomas and sarcomas we smoothed each individual tumor CGH profile using KC-SMART (kernel width: 20 Mb). For a range of thresholds, gains exceeding a positive threshold and losses exceeding the same negative threshold were added and averaged over each tumor group. Between KSE cutoffs of 0.14 and 0.4, the amount of CNAs is significantly different between carcinomas and sarcomas, calculated with a two sided t-test, P < 0.05, and shown by a gray background.

After stratifying the *p53*^*Δ/Δ *^tumors according to tumor type, we found that the carcinomas and adenomyoepitheliomas have significantly more genomic instability compared with sarcomas (Figure 3). This could explain why the KSE curves from the *p53*^*Δ/Δ *^tumor groups (which contain both carcinosarcomas and carcinomas) remain at lower level compared with the KSE curves from the *Brca1*^*Δ/Δ*^*;p53*^*Δ/Δ *^tumor and the *Brca1*^*Δ/Δ*^*;p53*^*Δ/Δ *^tumor groups which contains mainly carcinomas (Figure 1b).

### aCGH analysis of human breast tumors

**Figure 4:**
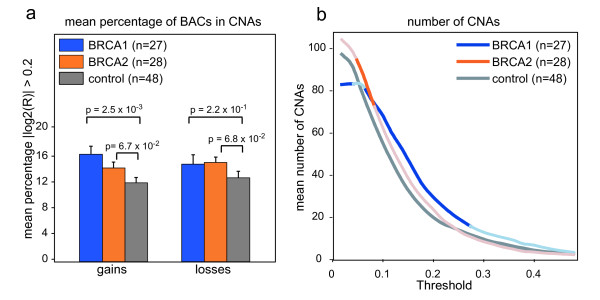
**Genomic instability of human BRCA1-mutated, BRCA2-mutated and sporadic control breast tumors**. **(a) **The mean percentage of BAC clones ± S.E.M. with absolute log2 ratio >0.2 is greater in across the 27 *BRCA1-*mutated tumors (blue) and 28 *BRCA2-*mutated tumors (orange) compared with 48 sporadic control tumors. P-values are determined by a two-tailed t-test with unequal variance. **(b) **Each individual tumor aCGH profile was smoothed using KC-SMART (kernel width: 20 Mb). For a range of thresholds, all gains exceeding a positive threshold and losses exceeding the same negative threshold were counted and averaged over each tumor group. Curves are darkened at thresholds for which the average number of CNAs per tumor group is significantly greater in the *BRCA1/2*-mutated tumors compared with the sporadic control tumors calculated with a two sided t-test with unequal variance, P < 0.05.

We compared aCGH data from our *Brca1*^*Δ/Δ*^*;p53*^*Δ/Δ*^, *Brca2*^*Δ/Δ*^*;p53*^*Δ/Δ *^and *p53*^*Δ/Δ *^tumor groups with aCGH data from previously published *BRCA1-*mutated, *BRCA2-*mutated and control human breast cancers [22,23]. First, we assessed the genomic instability of the human tumors by calculating the mean percentage of BAC clones with log2 ratio >0.2 for gains and <-0.2 for losses separately. The mean log2-ratio of the gains and the losses in the aCGH data of human tumors was lower compared to the mouse aCGH data (data not shown); hence, a lower cutoff was used to calculate the percentage of BACs reporting gains or losses in the human CNAs. This percentage was significantly higher for the gains of the *BRCA1-*mutated tumor group but not for the *BRCA2-*mutated tumor group when compared to the sporadic control tumors (p = 2.5 × 10^-3 ^calculated with t-test with unequal variance) (Figure 4a). The fraction of losses in the BRCA1 and BRCA2 tumor groups were not significantly different from The fraction of losses found in the sporadic control group. Next, to get an indication of the amount of CNAs in each tumor group, we smoothed the individual aCGH profiles with KC-SMART and determined the mean number of gains and losses in the KSE curves exceeding a range of thresholds. The *BRCA1-*mutated tumor group had significantly more CNAs compared with the sporadic control tumor group between thresholds of 0.08 - 0.24. The average amount of gains and losses in the *BRCA2-*mutated tumor group was not significantly increased compared with the control group except between thresholds of 0.06 and 0.08 (Figure 4b).

### Cross-species comparison of mouse and human breast tumors

Using aCGH data from genetically defined mouse mammary tumors as a data filter to mine the human breast cancer genome may be a powerful strategy to identify genomic regions with functional relevance for breast tumorigenesis [17]. To identify and narrow down regions with highest relevance for *BRCA *related tumor formation, we compared the array-CGH profiles of human *BRCA*-mutated and sporadic breast tumors with profiles from mouse BRCA-deficient and -proficient mammary tumors. First, we performed a cross species analysis using KC-SMART [18], which identifies significantly recurrent CNAs within each tumor group. Second, we adapted KC-SMART to *comparative*-KC-SMART which identifies CNAs that occur with significantly different frequency in one tumor group compared to another (see Methods). Next, we performed cross species analysis using *comparative-*KC-SMART, which identified regions that occurred in BRCA-mutated tumors but not in BRCA-proficient tumors (or vice versa) in both human and mouse breast tumors.

The following paragraphs describe the identification of cross-species syntenic gains and losses. However, they also show the challenges of interpreting cross-species genomic data.

#### Cross-species analysis using KC-SMART

**Figure 5:**
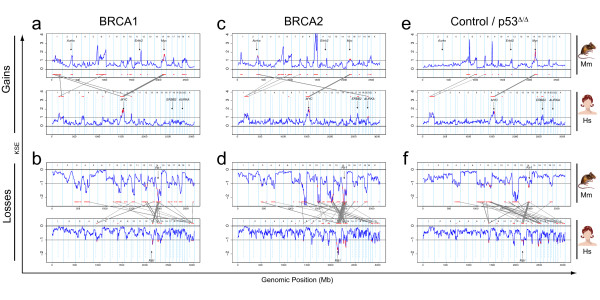
**Cross species KC-SMART analyses: overlapping recurrent CNAs of human and mouse breast tumors**. We analyzed aCGH data from mouse and human tumor groups with the KC-SMART algorithm using a kernel width of 20 Mb. Kernel Smoothed Estimate (KSE) curves are shown for gains and losses separately. For each human-mouse comparison, the upper section shows the KSE curve of the mouse tumor group (Mm) and the lower section shows the human tumor group (Hs). Regions that are gained or lost significantly more often compared to random are depicted in red above or below the KSE curves. Genes that map to a syntenic region of significant gain or loss are plotted in red on the KSE curves and are connected with gray lines between the species. The KSEs of the aCGH data were scaled such that the significance threshold determined by KC-SMART analysis was set at 1 for gains and at -1 for losses. **(a) **Top panel: the gains on the mouse *Brca1*^*Δ/Δ*^*;p53*^*Δ/Δ *^tumor group linked to the gains of the human BRCA1 tumor group (bottom panel). **(b) **Top panel: the losses on the mouse *Brca1*^*Δ/Δ*^*;p53*^*Δ/Δ *^tumor group linked to the losses of the human BRCA1 tumor group (bottom panel). **(c,d) **Idem: mouse *Brca2*^*Δ/Δ*^*;p53*^*Δ/Δ *^and human BRCA2 tumor groups **(e,f) **Idem: mouse *p53*^*Δ/Δ *^and the human control tumor groups. The genomic locations of *MYC*, *RB1*, *AURKA *and *ERBB2 *genes are shown. Cancer related genes that map in the overlapping regions are shown in Figure 6 (reduced list) and Additional file 6 (complete list).

**Figure 6:**
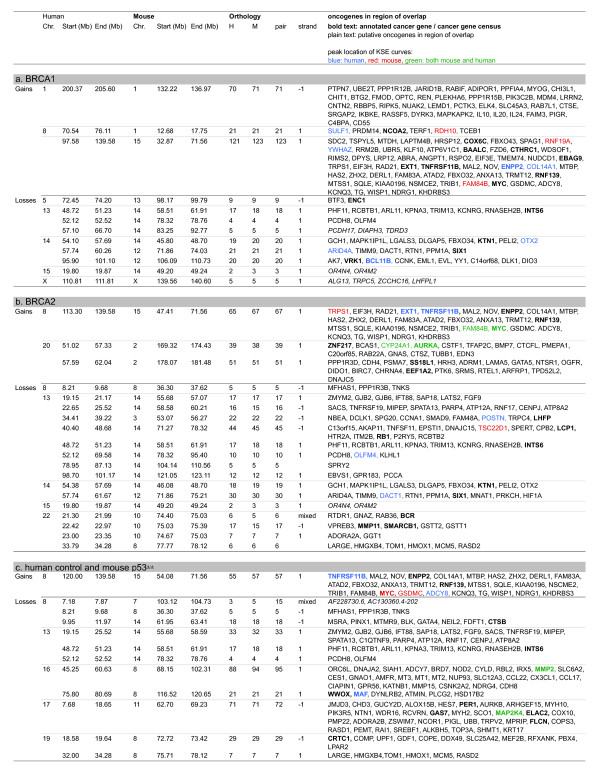
**Genes in overlapping recurrent CNAs of human and mouse breast tumor determined by cross species KC-SMART analyses**. Overview of genes in regions found by cross species KC-SMART analysis (Figure 5): listed are genes that map to significantly recurrent regions of gain and loss that overlap between (**a**) human *BRCA1*-mutated breast tumors and mouse *Brca1*^*Δ/Δ*^*;p53*^*Δ/Δ *^mammary tumors, (**b**) human *BRCA2*-mutated breast tumors and mouse *Brca2*^*Δ/Δ*^*;p53*^*Δ/Δ *^mammary tumors, (**c**) human control breast tumors and mouse *p53*^*Δ/Δ *^mammary tumors. The syntenic genomic regions on the human and the mouse genome are listed as well as the amount of mouse genes (M) and human genes (H) mapping within one region of overlap and the amount of unique orthologies for each pair of mouse and human genes (pair). The strand inversion between the two species (when more than one gene maps within the region): 1: no strand inversion, -1: strand inversion. Cancer related genes that map to the regions according to the Atlas of Genetics and Cytogenetics in Oncology and Haematology [44,45], and the Cancer Gene Census [46,47] are listed: annotated cancer genes and genes from the Cancer Gene Census are shown in bold type, putative cancer related genes are shown in normal type. Cancer related genes that map closest to the human KSE peak are shown in blue, genes that map closest to the mouse KSE peak are shown in red, and genes that map to the mouse AND human KSE peaks are shown in green. Overlapping regions with only one orthologue are not shown, for a complete list, including genes removed and added see Additional file 6.

We used the KC-SMART method [18] to identify significantly recurrent CNAs in mouse and human breast tumors and compared them between the two species (Figure 5). Regions of significant gain or loss determined by KC-SMART are shown in Additional file 4 for mouse tumors and in Additonal file 5 for human tumors. For comparison of mouse and human CNAs, we used a synteny list of human genes and their mouse orthologues (see Methods for details). Overlapping CNAs in our cross-species comparison, and the cancer-related genes that map within these regions are listed in Figure 6 (single overlapping genes were left out, for complete list see Additional file 6). We identified human and mouse KSE peak locations and depicted the closest cancer-related genes in Figure 6.

We identified one overlapping gain recurrent in all six tumor groups on human chromosome 8q (mouse chromosome 15). In all mouse tumor groups, the peak of this gain was located at (for *Brca2*^*Δ/Δ*^*;p53*^*Δ/Δ *^and *p53*^*Δ/Δ *^tumors) or near (for *Brca1*^*Δ/Δ*^*;p53*^*Δ/Δ *^tumors) the region containing the *MYC *oncogene at 128.8 Mb. The peak of the human *BRCA2-*mutated tumors mapped exactly on the *MYC *oncogene, and the peak of the human control tumor group maps slightly downstream of *MYC *(*ADCY8*, 132.21 Mb). All human tumor groups had a second peak on chromosome 8 mapping between *EXT1/TNFRSF11B *at 119.4 Mb and *ENPP2/COL14A1*, 120.87 Mb). This second peak was not shared with the mouse tumor groups.

In all tumor groups, we found a recurrent region of loss on chromosome 13q (mouse chromosome 14) which is commonly associated with the *RB1 *gene. Indeed, the overlapping regions of the mouse and human BRCA2 groups include *RB1*. The overlap of BRCA1 and control tumors mapped to the flanks of these peaks, notwithstanding that loss of *RB1 *could well drive the loss of this region in tumors of both species. Interestingly, the overlap of all three tumor groups included the region containing *INTS6*, an annotated tumor related gene (also known as *DICE1*, Protein deleted in cancer 1).

A remarkable similarity between mouse and human *BRCA2-*mutated breast tumors was that the peak of both the human (chromosome 20) and mouse (chromosome 2) centered exactly on the Aurora kinase A (*AURKA) *oncogene. Similarly, the human control tumors showed recurrent losses on chromosomes 16q and 17p, and the peaks of syntenic losses in mouse *p53*^*Δ/Δ *^tumors centered on exactly the same genes on chromosomes 8 (*MMP2*) and 11 (*MAP2K4*), respectively. This suggests that these genes (or neighboring genes) may be involved in selection of these CNAs during mouse and human tumor development. Note, however, that the *Aurka *gain also occurred in mouse *Brca1*^*Δ/Δ*^*;p53*^*Δ/Δ *^tumors and that loss of mouse chromosomes 8 and 11 was not specific for the *Brca1*^*Δ/Δ*^*;p53*^*Δ/Δ *^tumors, suggesting that BRCA2 association of the syntenic gain and BRCA1 association of the syntenic losses are dictated by the human tumor data.

Large regions of chromosome 1 were recurrently gained in the *Brca1*^*Δ/Δ*^*;p53*^*Δ/Δ *^tumors whereas shorter regions were gained in the *Brca2*^*Δ/Δ*^*;p53*^*Δ/Δ *^tumors (see Figure 2). Due to this difference in the mouse tumors, there was a BRCA1-specific overlap with human chromosome 1, as all other human tumor groups showed this significantly recurrent gain. It is interesting that the region of overlap encompasses *PIK3CB *and *MDM4*.

In the BRCA1 and BRCA2 tumor groups of both species, we identified a recurrent loss on human chromosome 14 (mouse chromosome 12), peaking on *ARID4A *(57.87 Mb) and *DACT1 *(58.17 Mb). All mouse tumors, including the *p53*^*Δ/Δ *^group, show this loss, encompassing entire chromosome 12. Whether the region containing *ARID4A *and *DACT1 *contains a driver gene for this whole chromosome loss remains to be established.

#### Cross species analysis using *comparative-*KC-SMART

**Figure 7:**
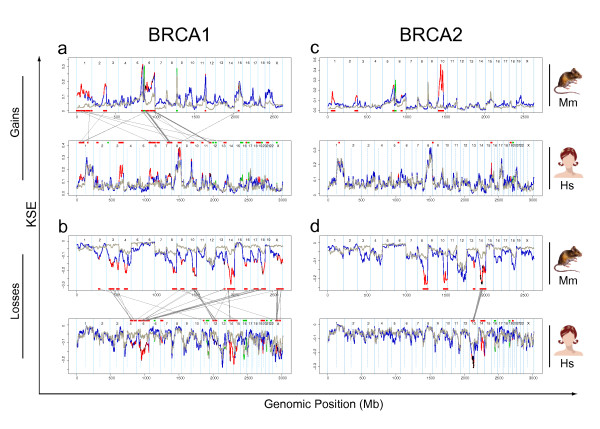
**Cross species comparative-KC-SMART analyses**. **(a) **The upper section shows the KSE curves of the gains of the mouse *Brca1*^*Δ/Δ*^*;p53*^*Δ/Δ *^(blue) and *p53*^*Δ/Δ *^(gray) tumor groups. The positions of the genes that map in regions that are aberrated significantly more often in the *Brca1*^*Δ/Δ*^*;p53*^*Δ/Δ *^compared to the *p53*^*Δ/Δ *^group are depicted in red on the blue curve and shown as red bars on the bottom of the panel. Vice versa, the positions of the genes that map in regions that are aberrated significantly more often in the *p53*^*Δ/Δ *^compared to the *Brca1*^*Δ/Δ*^*;p53*^*Δ/Δ *^group are depicted in green on the gray curve and shown as green bars on the bottom of the panel. Similarly, the lower section shows the KSE curves of the gains of the mouse human *BRCA1-*mutated (blue) and sporadic (gray) tumor groups. Those genes that map to syntentic regions that are differentially aberrated in tumors of both species are connected between the two plots by gray lines. Thicker connecting lines represent multiple genes that map to one syntenic region of gain. **(b) **Idem: differential losses of the mouse *Brca1*^*Δ/Δ*^*;p53*^*Δ/Δ *^(blue) and *p53*^*Δ/Δ *^tumor groups (gray) and human BRCA1-mutated (blue) and sporadic tumors(gray) (lower section). **(c,d) **Idem: differential gains and losses of the mouse *Brca2*^*Δ/Δ*^*;p53*^*Δ/Δ *^(blue) and *p53*^*Δ/Δ *^tumor groups (gray) and human BRCA2-mutated (blue) and sporadic tumors(gray) (lower section). Gains: No genes were differentially aberrated in tumors of both species. Cancer related genes that map in the overlapping regions are shown in Figure 8 (abbreviated list) and Additional file 9 (complete list).

To identify the CNAs that occur significantly more often in mouse BRCA1-deficient tumors compared to control tumors, we compared aCGH data from the mouse *Brca1*^*Δ/Δ*^*;p53*^*Δ/Δ *^tumors with aCGH data from the *p53*^*Δ/Δ *^tumors using *comparative-*KC-SMART. Similarly, to determine which CNAs occur significantly more often in the human *BRCA1-*mutated breast tumors compared to the control tumor group, we used *comparative-*KC-SMART on aCGH data from human tumors. Differentially occurring gains are depicted in Figure 7a and the differential losses are depicted in 7b; top panels represent mouse *Brca1*^*Δ/Δ*^*;p53*^*Δ/Δ *^tumors compared with *p53*^*Δ/Δ *^tumors and lower panels represent human *BRCA1*-mutated tumors compared to the control tumor group. We next determined which genomic regions are differentially recurrent in both mouse and human *BRCA1-*mutated tumors by mapping the orthologous genes of our synteny list to the differentially aberrated regions in both species. The mouse and human orthologues are connected by gray lines between the top panel and the lower panel. We applied the same analysis to the mouse and human *BRCA2-*mutated tumors (Figure 7c,d).

**Figure 8:**
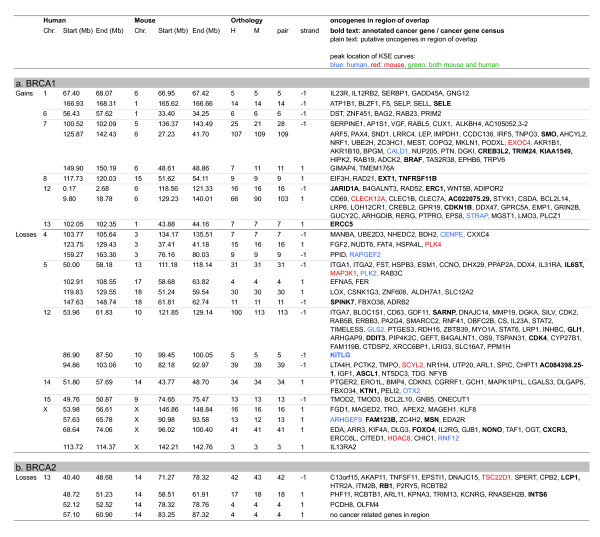
**Genes in overlapping recurrent CNAs of human and mouse breast tumor determined by cross species comparative-KC-SMART analyses**. Overview of genes in regions found by cross species *comparative-*KC-SMART analysis (Figure 7). Listed are genes that map to significantly differentially gains and losses that overlap (**a**) between mouse *Brca1*^*Δ/Δ*^*;p53*^*Δ/Δ *^mammary tumors (compared to *p53*^*Δ/Δ *^tumors) and human *BRCA1-*mutated breast tumors (compared to control tumors) and (**b**) between mouse Brca2^Δ/Δ^;p53^Δ/Δ ^mammary tumors (compared to p53^Δ/Δ ^tumors) and *BRCA2-*mutated breast tumors (compared to control tumors). Idem Figure 6. For complete list see Additional file 9.

Regional CNAs that occur significantly more often in the *Brca1*^*Δ/Δ*^*;p53*^*Δ/Δ *^or *Brca2*^*Δ/Δ*^*;p53*^*Δ/Δ *^mouse tumor groups compared with the *p53*^*Δ/Δ *^control tumors are listed in Additional file 7. For the complementing human data see Additional file 8. The regions of human-mouse overlap determined by *comparative-*KC-SMART are listed in Figure 8 (regions with single overlapping genes were left out, for complete list see Additional file 9). Between the human and mouse, control tumors show no syntenic regions of overlap in any of the comparisons, therefore, the orthologous genes mapping to these regions are not connected. Regarding regions encompassing more than one gene, there are 26 regions that overlap between mouse and human *BRCA1-*mutated tumors (10 overlapping BRCA1-specific gains and 16 losses), compared to only 4 overlapping BRCA2-specific losses. This marked difference is due to the lack of differential gains and losses between the human BRCA2 tumors and sporadic control tumors.

The largest overlapping BRCA1-specific gain between mouse and human *BRCA1-*mutated breast tumors maps to human chromosome 1 and to mouse chromosome 6. This region harbors the *GADD45A *gene, shown to be induced by BRCA1 independently of p53 [25], and implicated in regulating centrosome duplication and maintaining genome integrity [26]. We speculate that the GADD45A gene cannot be induced in BRCA1-deficient cells, and that the necessity for its activity during cellular division could select for its expression by amplification.

The gain on human chromosome 7 maps to mouse chromosome 6. This is one of the few gains in the human tumors that are seen in the *BRCA1-*mutated tumors but not in the control tumors. Similarly, the gain in the centromeric region of mouse chromosome 6 is also not seen in the *p53*^*Δ/Δ *^tumors, suggesting that in this setting, this gain is BRCA1-specific in tumors of both species. The mouse tumors have a peak on the *EXOC4 *gene (alias *SEC8*, at 132.9 Mb), and the human peak at the *CALD1 *gene (Caldesmon 1, at 134.2 Mb). SEC8 is involved in cell-cell adhesion and is essential for the development of epithelial cell surface polarity. Caldesmon is a potential actomyosin regulatory protein found in smooth muscle and nonmuscle cells. Whether amplification of these genes selects for tumor development remains speculation. There are several interesting cancer-mutated genes in this region such as *SMO *(smoothened, at 128.63 Mb) which was previously associated with basal-like carcinomas [27] and the *BRAF *oncogene (140.17 Mb) which regulates proliferation differentiation, senescence and apoptosis through the RAS/ERK pathway. Since these two genes roughly mark the edges of the common amplicon it remains unclear whether these genes are involved in driving the development of this gain.

The overlapping gain on the telomeric end human chromosome 12p maps to the telomeric part of mouse chromosome 6. The mouse tumors have a small peak on *CLEC12A*, but the actual peak of the amplicon is at the end of chromosome 6, which encompasses the oncogenic *Kras *gene; however, this region is not part of the human-mouse overlap. The human tumors have a peak on *STRAP *(alias *MAWD*, at 15.93 Mb) which has previously been shown to be overexpressed in breast cancer [28]. The driving gene(s) of this overlapping gain might therefore differ between mouse and human breast tumors.

Loss of human chromosome 5q is strongly associated with *BRCA1-*mutated tumors [29]. This region might therefore contain tumor suppressor gene(s) that collaborate with BRCA1-loss in tumorigenesis. The first part of human chromosome 5q maps to the telomeric half of mouse chromosome 13, which is not lost significantly more often in the mouse *Brca1*^*Δ/Δ*^*;p53*^*Δ/Δ *^tumors compared to the *p53*^*Δ/Δ *^tumors, with the exception of the telomeric region. The human peak (which is only a small peak adjacent to a more intense peak) and the mouse peak localize at the region encompassing the *MAP3K1 *and *PLK2 *genes. There are two chromosome 5 syntenies mapping to consecutive regions on mouse chromosome 18. The *SPINK7 *gene (also known as *ECRG2*, esophageal cancer related gene 2) maps close to the peak of both the mouse and human loss. Again, its involvement in driving the chromosome 5 loss remains to be established.

The BRCA1-specific loss on human chromosome 12 maps to mouse chromosome 10 and consists of three separate losses. The first part peaks at the *GLS2 *gene, whereas the mouse loss peaks at the *ITGA7 *gene. This loss contains several interesting genes such as *CD63 *(melanoma-associated antigen), *CDK2*, *ERBB3*, *CDK4 *and the *DDIT3*, but since these genes are part of the mitogenic signaling pathways, it is unlikely that these genes are drivers of this loss. The second loss on human chromosome 12 is a peak region encompassing five genes, of which one, *KITLG*, is listed as a putatively cancer related gene. The third loss on chromosome human 12 also maps to mouse chromosome 10. The mouse loss peaks on *APAF1*, involved in apoptosis, and the human tumors show a peak at the end of the loss, just outside the overlap.

The human *BRCA2-*mutated tumor group is very comparable to the control tumor group, and therefore shows only one overlapping region with mouse *Brca2*^*Δ/Δ*^*;p53*^*Δ/Δ *^tumors. The human chromosome 13 is one consecutive loss previously associated with *RB1*, which maps in different parts to mouse chromosome 14. Indeed, *RB1 *(47.95 Mb) maps to this loss, close to the peaks of both mouse (*TSC22D1 *at 43.9 Mb) and human tumors (*OLFM4 *at 52.5 Mb).

## Discussion

During tumor development, acquired genetic aberrations that confer selective advantage will become enriched in the tumor cell population. To identify the drivers underlying the gains and losses selected for during breast cancer development, we made use of the synteny between human and mouse genomes [30]. Syntenic gains and losses that occur in both the mouse and human oncogenomes may harbor evolutionary conserved genes or genetic elements that are causal to tumor development in both species. Indeed, comparative aCGH analyses showed that many genetic aberrations are conserved between human and mouse neuroblastomas [31,32], ovarian cancers [33], hepatocellular carcinomas [34], melanomas [35] and T-cell lymphomas [36]. In this work, we analyzed and compared aCGH data from a large series of mouse and human *BRCA1- *and *BRCA2-*mutated breast cancers, in order to obtain a complete collection of genes and loci that are recurrently gained or lost in tumors of both species. We find that amplification of the MYC locus and the loss of the RB/INTS6 locus is evolutionarily conserved in mouse and human mammary tumor development, and that *AURKA *amplification is conserved in mouse and human *BRCA2-*mutated tumors. However, aside from these similarities, we also find recurrent aberrations in mouse and human oncogenomes that do not overlap.

### Brca1 and Brca2 loss intensifies aCGH patterns of mouse *p53^Δ/Δ ^*mammary tumors

Because mouse models enable targeted deletion of specific gene(s) in a defined genetic background it is possible to assess the specific function of this gene within an organism. With our mouse models, we analyzed the impact of BRCA1 or BRCA2 loss on the oncogenome, in a p53 deficient background. KC-SMART profiles of mouse *Brca1*^*Δ/Δ*^*;p53*^*Δ/Δ *^and *Brca2*^*Δ/Δ*^*;p53*^*Δ/Δ *^mammary tumors showed that significantly recurrent genomic aberrations largely overlap with recurrent aberrations in *p53*^*Δ/Δ *^tumors and that these common aberrations occur more frequently in *Brca1*^*Δ/Δ*^*;p53*^*Δ/Δ *^and *Brca2*^*Δ/Δ*^*;p53*^*Δ/Δ *^mammary tumor compared to the *p53*^*Δ/Δ *^tumor group. This suggests that BRCA1 or BRCA2 loss intensifies the profile of aberrations seen in the *p53*^*Δ/Δ *^tumor group.

We find that the *p53*^*Δ/Δ *^tumors that are BRCA proficient more often gain or lose entire chromosomes during tumor development. In contrast, *Brca1*^*Δ/Δ*^*;p53*^*Δ/Δ *^and *Brca2*^*Δ/Δ*^*;p53*^*Δ/Δ *^tumors have an increased amount of partial chromosomal gains and losses, presumably because they have accumulated more inter- and intrachromosomal DNA damage as a result of error-prone DSB repair in the absence of homology directed DNA repair. The inability of these cells to properly repair DSBs might allow selection of gains and losses more specifically tailored around the relevant genes in a region. Interestingly, most gains and losses found in the *p53*^*Δ/Δ *^tumor group are intensified by *Brca1 *or *Brca2 *loss but some are not, such as the gain on chromosome 15 and the losses on chromosomes 7 and 12. These regions may therefore be subject to a differential selection pressure during tumor development.

It appears that cell type also plays an important role: the carcinomas of the *p53*^*Δ/Δ *^tumor group seems to have more genomic damage compared with the carcinosarcomas. This may be the reason that BRCA1 and BRCA2 loss gives rise to a more aggravated DNA damage profile, as >90% of *Brca1*^*Δ/Δ*^*;p53*^*Δ/Δ *^tumors and *Brca2*^*Δ/Δ*^*;p53*^*Δ/Δ *^mammary tumors are carcinomas, compared to half of the *p53*^*Δ/Δ *^mammary tumors, of which the other half are sarcomatoid. This suggests that in these mouse models BRCA1 loss and BRCA2 loss blocks cellular differentiation as suggested previously [37], or alternatively, that BRCA1 and BRCA2 loss are not tolerated in more differentiated cells.

### Differences between mouse Brca1 and Brca2 mutated tumors

Despite their common p53 deficient background, some aberrations may be more prone to arise in a BRCA1 deficient cell, whereas others are more likely to occur in a BRCA2 deficient cell. For example, the recurrent gain on chromosome 1 or the losses on chromosomes 4 and 9 of the *Brca1*^*Δ/Δ*^*;p53*^*Δ/Δ *^tumors are less common in the *Brca2*^*Δ/Δ*^*;p53*^*Δ/Δ *^tumors. Likewise, the prominent gain on chromosome 10 is found in half of all *Brca2*^*Δ/Δ*^*;p53*^*Δ/Δ *^tumors, and in approximately 1/8 of the *Brca1*^*Δ/Δ*^*;p53*^*Δ/Δ *^tumors. In addition, it appears that in a p53 deficient background, *Brca1 *loss gives rise to more genomic aberrations than does *Brca2 *loss. Together, these differences in tumor profiles suggest that BRCA1 and BRCA2 have different functions in DNA repair pathways, however, we have not been able to identify the nature of this difference using our aCGH profiles.

### Comparative oncogenomics identifies similarities and differences in mouse and human breast tumors

Cross species comparison of genomic aberrations in human and mouse tumors may identify conserved genomic regions likely to be important for development in both species. Tumors from genetically engineered mouse models often do not show the high levels of chromosome instability associated with human cancers [36], presumably because the engineered mutations predispose to rapid tumor formation and thereby circumvent the long incubation time required for accumulation of genomic mutations in human tumorigenesis. The increased amounts of genomic alterations in tumors from mice with engineered telomere dysfunction or defects in DNA damage checkpoints or DNA repair allows comparison of genomic alterations with human tumors that would not be possible in mouse models without chromosomal instability [36].

In our analysis, we compared aCGH profiles from human *BRCA1/2*-mutated and sporadic breast cancers with profiles from mouse *p53*^*Δ/Δ*^, *Brca1*^*Δ/Δ*^*;p53*^*Δ/Δ *^and *Brca2*^*Δ/Δ*^*;p53*^*Δ/Δ *^mammary tumors. We assume that DNA damage repair deficiency in the mammary epithelium of these mice allows a comparable selective advantage in tumor formation as in human breast epithelial cells. Indeed, all human tumor groups showed a recurrent gain on chromosome 8q, likewise all mouse tumor groups showed a recurrent gain on its syntenic region, on chromosome 15, centering exactly on the *MYC *oncogene. This suggests that *MYC *gain is evolutionarily conserved in mammary tumor development from mouse to man. Likewise, the chromosome 13 loss, associated with RB1 loss [38] occurs in all human and mouse tumor groups, and all overlapping regions encompass the *INTS6 *gene. Similar to *MYC *gain, *RB1/INTS6 *loss could be evolutionarily conserved in mammary tumor development.

The defined initiating lesions and similar genetic background of mouse mammary tumors permits identification of recurrent genomic aberrations linked to certain genetic predispositions. Indeed, the amplification peak on human chromosome 20 overlapped with the syntenic amplification peak on mouse chromosome 2 of the *Brca1*^*Δ/Δ*^*;p53*^*Δ/Δ *^and the *Brca2*^*Δ/Δ*^*;p53*^*Δ/Δ *^tumors, at the *Aurora Kinase A *gene. The *AURKA *gain has been previously associated with *BRCA2-*mutated breast tumor development [39] but our findings suggest that the functional interaction between AURKA and BRCA2 may be evolutionarily conserved. The mouse *Brca1*^*Δ/Δ*^*;p53*^*Δ/Δ *^tumors also showed the gain on chromosome 2 but the *p53*^*Δ/Δ *^tumors, on the other hand, did not show this gain at all, suggesting that this gain may selectively develop in a homologous repair deficient (HRD) background.

For most other overlapping gains and losses reported in the results section, it is still difficult to associate a driver gene with a gain or a loss. Also, gains known to be important in human *BRCA1-*mutated breast cancer show no (e.g. 3q gain) or only partial (e.g. the 5q loss) overlap with the mouse gains and losses. The human 3q gain is associated with *PIK3CA *overexpression; possibly, mouse tumors may find alternative ways to overexpress this gene or a functional homologue. Alternatively, *PIK3CA *overexpression might not provide a selective advantage in these mouse tumors, or mouse tumors in general. The regions susceptible for amplification or deletion in the mouse genome and the human genome may not be the same due to, for example, different nuclear chromosomal folding or genomic organization of synteny blocks, resulting in a differential development of the oncogenome during mouse and human tumor development. These findings add to previous findings of differences between mouse and human breast cancer development such as that activation of Wnt and/or its pathway members are commonly found in mouse but not in human spontaneous breast tumors, differences in metastasis patterns, or that few mouse models accurately represent ER positive human breast cancers [40].

## Conclusions

In this work we have found some important genotypic similarities between mouse and human cognate cancers, such as the *MYC *gain and the *RB/INTS6 *loss, but also important differences such as the chromosome 10 gain in mouse *Brca2*^*Δ/Δ*^*;p53*^*Δ/Δ *^tumors and the 3q gain in human *BRCA1-*mutated tumors. In our mouse models, we find that BRCA1 and BRCA2 loss clearly have different impacts on tumor development. However, the distinct features of mouse *Brca1*^*Δ/Δ*^*;p53*^*Δ/Δ *^and *Brca2*^*Δ/Δ*^*;p53*^*Δ/Δ *^tumors did not translate to distinct features of human *BRCA1 *and *BRCA2 *mutated tumors and vice versa. A reason for this could be that tumor development in our mouse models is a fast, accelerated version of the relatively slow process of tumor development in humans. Simultaneous and homozygous deletion of the mouse *Brca1*, *Brca2 *and *p53 *genes leads to immediate cellular transformation, whereas human germline *BRCA1 *and *BRCA2 *mutations are initially heterozygous [41,42]. Human *TP53 *mutations are somatic and can be acquired in myriad different ways, possibly resulting in different phenotypes [43]. In addition, the difference in make-up of the mouse and human genome, such as the genome wide organization of syntenic regions, and/or overall differences between mouse and human mammary gland biology may result in selection of other regions and genes during the course of mouse and human breast cancer development. These differences should be taken into account when using *K14cre*;*Brca1*^F/F^;*p53*^F/F ^and *K14cre*;*Brca2*^F/F^;*p53*^F/F ^mouse models for preclinical studies.

## List of Abbreviations

aCGH: array comparative genomic hybridization; CNA: (genomic) copy number alterations; DSB: double strand break; ER: Estrogen Receptor; KC-SMART: Kernel Convolution - a Statistical Method for Aberrant Region detection; GEM: genetically engineered mouse; IDC-nos: invasive ductal carcinoma not otherwise specified; KSE: Kernel Smoothed Estimate; BAC: Bacterial Artificial Chromosome; HRD: homologous recombination deficient; SNR: signal to noise ratio; FDR: False Discovery Rate

## Competing interests

The authors declare that they have no competing interests.

## Authors' contributions

HH, EvB, PN and JJ conceived and designed the study. HH, EvB, AV, XL, SAJ, SK, ES, RK and JJ performed experiments. HH, EvB, PN and JJ analyzed the data. CNK, AV and LFAW contributed analysis tools. HH, EvB, PN and JJ wrote the paper. All authors read and approved the final manuscript.

## Pre-publication history

The pre-publication history for this paper can be accessed here:

http://www.biomedcentral.com/1471-2407/10/455/prepub

## Supplementary Material

Additional file 1 **Histopathology, BRCA1/BRCA2 mutations, tumor types of human breast tumors**. Immunohistochemistry: Presence of ER, PR, ERBB2 (HER2/neu) and TP53 was determined by immunohistochemistry staining using the antibodies: estrogen receptor AB-14 clone 1D5 + 6F11, titre 1:50 (Neomarkers); progesterone receptor clone PR-1 titre 1:400 (Immunologic), c-erbB-2 clone SP3, titre 1:25 (Neomarkers); TP53 clone D0-7, titre 1:8000 (Dako). If >70% of the tumor cells expressed ER, PR, or TP53, the tumor was scored as positive (+) for the corresponding staining, in case <10% of the cells were stained, the tumor was scored as negative (-) and between 10 and 70% the tumor was scored as ± for the corresponding staining. HER2/neu staining was scored positive when a 3+ staining was observed (*), otherwise it was scored negative (only one sporadic control case was IHC 2+, and was called negative). *) Wolff AC, Hammond ME, Schwartz JN, Hagerty KL, Allred DC, Cote RJ, et al. American Society of Clinical Oncology/College of American Pathologists guideline recommendations for human epidermal growth factor receptor 2 testing in breast cancer. J Clin Oncol 2007 Jan 1;25(1):118-45. Click here for file

Additional file 2 **Tumor type characterization of mouse mammary tumors by Vimentin and E-Cadherin expression analysis**. To determine tumor type, log2 ratios of Vimentin and E-Cadherin expression were acquired by hybridization of mRNA of a subset of tumors on Mouse Operon V3 Oligo arrays and were plotted against each other. a) *Brca1*^*Δ/Δ*^*;p53*^*Δ/Δ*^, *Brca2*^*Δ/Δ*^*;p53*^*Δ/Δ *^and *p53*^*Δ/Δ *^mouse tumors were plotted by their E-cadherin and Vimentin expression levels. **b) **If the difference between E-cadherin and Vimentin expression levels was less than 0.5 on a log scale these tumors were called 'ambiguous'. Tumors were scored 'Mesenchymal' if the Vimentin expression level was >0.5 higher than E-cadherin expression level. Likewise, tumors were scored 'Epithelial' when the E-cadherin expression level was >0.5 higher than the Vimentin expression. Click here for file

Additional file 3 **Tumor types of mouse mammary tumors determined by histopathology and gene expression analysis**. Mouse mammary tumor types were determined by histopathology and expression data. Histopathology was done as previously described in [10]. Expression data was acquired by hybridization of mRNA of a subset of tumors on Mouse Operon V3 Oligo arrays: If the difference between E-cadherin and Vimentin expression levels was less than 0.5 on a log scale we called these tumors 'ambiguous'. Tumors were scored 'Mesenchymal' if the Vimentin expression level was >0.5 higher than E-cadherin expression level. Likewise, tumors were scored 'Epithelial' when the E-cadherin expression level was >0.5 higher than the Vimentin expression level. The line numbers relate to the line-plots shown in Figure 2, line number 1 on top for each tumor group. Click here for file

Additional file 4 **KC-SMART analysis of mouse mammary tumors**. The significant CNAs of *Brca1*^*Δ/Δ*^*;p53*^*Δ/Δ*^, *Brca2*^*Δ/Δ*^*;p53*^*Δ/Δ *^and *p53*^*Δ/Δ *^mouse tumor groups were obtained by running the KC-SMART algorithm over the BAC data from all tumors in each tumor group using a kernel width of 20 Mb. Significant regions are determined by the intercept of the KSE curve and the significance cutoff calculated for each tumor group, and for gains and losses separately. The upper panel lists recurrent gains and the bottom panel lists the recurrent losses. These regions correspond to the color-matched bars on top (gains) and on the bottom (losses) of the KSE curves of the tumor groups shown in Figure 1d and the red bars of the cross species comparison in Figure 5. Click here for file

Additional file 5 **KC-SMART analysis of human breast tumors**. Genomic regions found recurrently gained (top) or lost (bottom) by KC-SMART analysis of human BRCA1-related, BRCA2-related, and control breast tumors. The significant CNAs were determined by running the KC-SMART algorithm over the BAC data from all tumors in each tumor group using a kernel width of 20 Mb. Significant regions are determined by the intercept of the KSE curve and the significance cutoff calculated for each tumor group, and for gains and losses separately. The upper panel lists recurrent gains and the bottom panel lists the recurrent losses. Click here for file

Additional file 6 **Regions identified by cross-species KC-SMART analysis**. Shown are genes within significantly recurrent regions of gain and loss that overlap between (**a**) human *BRCA1*-mutated and mouse *Brca1*^*Δ/Δ*^*;p53*^*Δ/Δ *^tumors, (**b**) human *BRCA2*-mutated and mouse *Brca2*^*Δ/Δ*^*;p53*^*Δ/Δ *^tumors, and (**c**) human control tumors and mouse *p53*^*Δ/Δ *^tumors as shown in Figure 5. The syntenic regions in the human and the mouse genome are listed in columns 1 and 2. The "Orthology" column lists the number of mouse (M) and human (H) genes mapping within each region of overlap, and the number of unique orthologous pairs (pair). Strand inversion between the two species is indicated by 1 (no inversion) or -1 (inversion). Listed are cancer-related genes included in the Atlas of Genetics and Cytogenetics in Oncology and Haematology [44,45], and the Cancer Gene Census (CGC)[47,46]. Annotated cancer genes and CGC genes are shown in bold type, putative cancer genes are shown in normal type. Cancer-related genes that map closest to the human KSE peak are shown in blue and genes that map closest to the mouse KSE peak are shown in red. Genes that map to the mouse AND human KSE peaks are shown in green. Single genes that were not listed in the Atlas or the CGC are shown in italics. Click here for file

Additional file 7 **Comparative-KC-SMART analysis of mouse mammary tumors**. The significant differential gains and losses of *Brca1*^*Δ/Δ*^*;p53*^*Δ/Δ *^vs. *p53*^*Δ/Δ *^and *Brca2*^*Δ/Δ*^*;p53*^*Δ/Δ *^vs. *p53*^*Δ/Δ *^mouse tumor groups were obtained by running the *comparative-*KC-SMART algorithm over the BAC data from all tumors in each tumor group using a kernel width of 20 Mb. The upper panel shows recurrent differential gains and the bottom panel shows the differential losses. These regions correspond to the red bars plotted on top (gains) and on the bottom (losses) of the corresponding mouse KSE curves used in the cross species comparison in Figure 6. Click here for file

Additional file 8 **Comparative-KC-SMART analysis of human breast tumors**. Comparative KC-SMART analysis of human BRCA1-related and BRCA2 related breast tumors compared with control breast tumors. Regions of differential aberrations between BRCA1 and control breast tumors, between BRCA2 and control breast tumors. Top panel: Differential gains. Bottom panel: Differential losses. Click here for file

Additional file 9 **Regions identified by cross-species *comparative*-KC-SMART analysis**. Shown are **(a) **genes that map to differentially recurrent CNAs found in both mouse *Brca1*^*Δ/Δ*^*;p53*^*Δ/Δ *^vs. *p53*^*Δ/Δ *^mammary tumors and human *BRCA1-*mutated vs. control breast tumors and (**b**) genes mapping to differentially recurrent CNAs found in both mouse *Brca2*^*Δ/Δ*^*;p53*^*Δ/Δ *^vs. *p53*^*Δ/Δ *^mammary tumors and *BRCA2-*mutated vs. control breast tumors, shown in Figure 7 Click here for file
