# Supplementary material for: Cross-species comparison of aCGH data from mouse and human BRCA1- and BRCA2-mutated breast cancers
Source: BMC Cancer. 2010 Aug 24;10:455. doi: 10.1186/1471-2407-10-455 (PMC2940799; doi:10.1186/1471-2407-10-455)
Supplement: Additional file 1 — Histopathology, BRCA1/BRCA2 mutations, tumor types of human breast tumors. Immunohistochemistry: Presence of ER, PR, ERBB2 (HER2/neu) and TP53 was determined by immunohistochemistry staining using the antibodies: estrogen receptor AB-14 clone 1D5 + 6F11, titre 1:50 (Neomarkers); progesterone receptor clone PR-1 titre 1:400 (Immunologic), c-erbB-2 clone SP3, titre 1:25 (Neomarkers); TP53 clone D0-7, titre 1:8000 (Dako). If >70% of the tumor cells expressed ER, PR, or TP53, the tumor was scored as positive (+) for the corresponding staining, in case <10% of the cells were stained, the tumor was scored as negative (-) and between 10 and 70% the tumor was scored as ± for the corresponding staining. HER2/neu staining was scored positive when a 3+ staining was observed (*), otherwise it was scored negative (only one sporadic control case was IHC 2+, and was called negative). *) Wolff AC, Hammond ME, Schwartz JN, Hagerty KL, Allred DC, Cote RJ, et al. American Society of Clinical Oncology/College of American Pathologists guideline recommendations for human epidermal growth factor receptor 2 testing in breast cancer. J Clin Oncol 2007 Jan 1;25(1):118-45. [file 1471-2407-10-455-S1.PDF]

BRCA1-related breast tumors, previously described in Joosse et al 2008 (1)					
NKI ID	p53 IHC	ER IHC	PR IHC	HER2/Neu IHC	BRCA1 mutation
B1007	0	0	0	0	c.1319delT
B1009	0	0	0	0	c.IVS21-36del510
B1016	1	0	0	0	c.185delAG
B1018	0	0	0	0	c.4416_4417delTTinsG
B1019	0	0	0	0	c.4416_4417delTTinsG
B1022	0	0	0	0	c.4446C>T
B1024	1	0	0	0	c.3875del4
B1025	0	0	0	0	c.2804delAA
B1026	1	0	0	0	c.IVS21-36del510
B1027	1	0	0	0	c.5382insC
B1035	1	0	0	0	c.2312del5
B1037	0	0	0	0	c.IVS12-1632del3835
B1041	0	0	0	0	c.IVS21-36del510
B1045	0	0	0	0	c.185delAG
B1046	0	0	0	0	c.185delAG
B1049	1	0	0	0	c.IVS20+1G>A
B1050	0	0	0	0	c.IVS21-36del510
B1052	1	0	0	0	c.IVS13+4123ins6081
B1053	1	0	0	0	c.185delAG
B1056	1	0	0	0	c.5382insC
B1058	1	0	0	0	c.IVS21-36del510
B1060	0	0	0	0	c.IVS20+1G>A
B1061	1	0	0	0	c.IVS13+4123ins6081
B1062	0	0	0	0	c.IVS20+1G>A
B1064	0	0	0	0	c.del exons 1A-7
B1065	1	0	0	0	c.IVS12-1632del3835
B1071	0	0	0	1	c.IVS2-9C>G

BRCA2-related breast tumors, previously described in Joosse et al, 2010 (2)					
NKI ID	p53 IHC	ER IHC	PR IHC	HER2/Neu IHC	BRCA2 mutation
B2004	0	1	0	0	c.5946delT, p.Ser1982fs
B2007	0	1	0	0	c.6275_6276delTT, p.Leu2092fs
B2010	0	0	0	1	c.5946delT, p.Ser1982fs
B2011	0	1	0	0	c.6275_6276delTT, p.Leu2092fs
B2014	0	1	1	0	c.9099_9100delTC, p.Thr3033fs
B2015	0	1	1	0	c.5681dupA, p.Tyr1894fs
B2017	0	1	0	1	c.5681dupA, p.Tyr1894fs
B2023	0	1	1	1	c.115delG, p.Ala39fs
B2025	1	1	0	0	c.5564C>A, p.Ser1855X
B2028	1	0	0	0	c.5722_5723delCT, p.Leu1908fs
B2030	1	1	1	0	c.6275_6276delTT, p.Leu2092fs
B2034	1	1	1	0	5573insA exon 11
B2040	1	1	1	0	c.1796_1800del5, p.Phe599fs
B2041	NA	NA	NA	NA	6819delTG
B2045	1	1	0	0	c.6275_6276delTT, p.Leu2092fs
B2050	NA	NA	NA	NA	c.6275_6276delTT, p.Leu2092fs
B2051	NA	NA	NA	NA	c.5722_5723delCT, p.Leu1908fs
B2052	0	1	0	0	c.8067T>A, p.Cys2689X
B2053	NA	NA	NA	NA	6648insA exon 11
B2055	1	1	0	0	6503delTT exon11
B2057	1	0	0	0	c.6644_6647del4, p.Tyr2215fs
B2060	0	1	0	0	c.6275_6276delTT, p.Leu2092fs
B2062	0	1	1	0	c.8067T>A, p.Cys2689X
B2067	1	1	0	0	c.9025delT, p.Tyr3009fs
B2070	NA	NA	NA	NA	NA
B2073	NA	NA	NA	NA	NA
B2075	NA	NA	NA	NA	NA
B2077	NA	NA	NA	NA	NA

Sporadic control tumors, previously described in Joosse et al 2008 (1)					
NKI ID	p53 IHC	ER IHC	PR IHC	HER2/Neu IHC	cell type
C001	0	0	1	0	HER2+/ER-
C002	0	0	0	1	Luminal A
C004	1	0	0	1	Basal-like
C006	0	0	0	1	HER2+/ER-
C010	0	1	1	0	Luminal A
C013	1	0	1	1	HER2+/ER-
C015	1	1	0	1	HER2+/ER-
C016	0	1	1	0	Normal Breast-like
C017	0	1	1	0	Luminal B
C018	0	0	0	0	Basal-like
C019	0	1	1	0	Basal-like
C020	1	0	0	0	Luminal A
C022	0	1	0	0	Luminal B
C023	0	1	1	1	HER2+/ER-
C025	0	1	1	0	Luminal A
C026	0	1	1	0	Normal Breast-like
C027	0	1	1	1	Luminal A
C028	0	1	1	0	Luminal B
C029	0	0	0	1	HER2+/ER-
C030	0	1	0	0	Luminal B
C031	1	0	0	1	HER2+/ER-
C032	0	1	1	0	Normal Breast-like
C033	1	0	0	0	Basal-like
C034	0	1	1	0	Luminal A
C035	0	NA	NA	1	NA
C036	0	1	1	0	Luminal A
C037	0	1	1	0	Luminal B
C039	0	NA	1	0	HER2+/ER-
C042	0	1	1	0	Luminal B
C043	0	0	0	1	HER2+/ER-
C044	0	1	0	0	Luminal B
C046	1	0	0	0	Basal-like
C047	1	0	0	0	Basal-like
C048	1	0	0	1	HER2+/ER-
C049	1	0	0	0	Basal-like
C051	0	0	0	1	HER2+/ER-
C052	0	1	1	0	Luminal A
C053	1	0	0	0	Basal-like
C056	1	0	0	0	Basal-like
C057	0	0	0	0	Luminal B
C058	1	1	0	1	HER2+/ER-
C060	1	1	1	0	Luminal B
C061	1	1	1	1	HER2+/ER-
C063	1	1	1	0	NA
C065	1	0	0	0	NA
C067	1	1	1	1	NA
C068	1	1	0	1	NA
C069	1	1	0	0	NA

1) Joosse SA, van Beers EH, Tielen IH, Horlings H, Peterse JL, Hoogerbrugge N, Ligtenberg MJ, Wessels LF, Axwijk P, Verhoef S *et al*: Prediction of BRCA1-association in hereditary non-BRCA1/2 breast carcinomas with array-CGH. *Breast Cancer Res Treat* 2008.

2) Joosse SA, Brandwijk KIM, Devilee P, Wesseling J, Hogervorst FBL, Verhoef S, Nederlof PM: Prediction of BRCA2-association in hereditary breast carcinomas using array-CGH. *Breast Cancer Res Treat* 2010, Epub ahead of print.
