# Supplementary figures and images for: Cross-species comparison of aCGH data from mouse and human BRCA1- and BRCA2-mutated breast cancers
Source: BMC Cancer. 2010 Aug 24;10:455. doi: 10.1186/1471-2407-10-455 (PMC2940799; doi:10.1186/1471-2407-10-455)

### Expression of E-cadherin and Vimentin in mouse mammary tumors

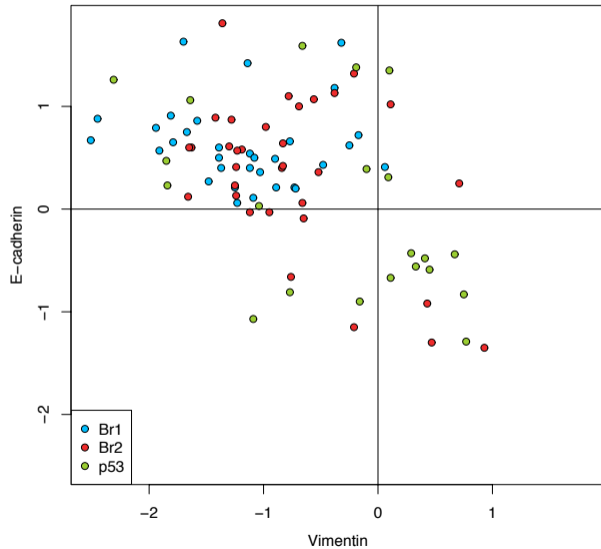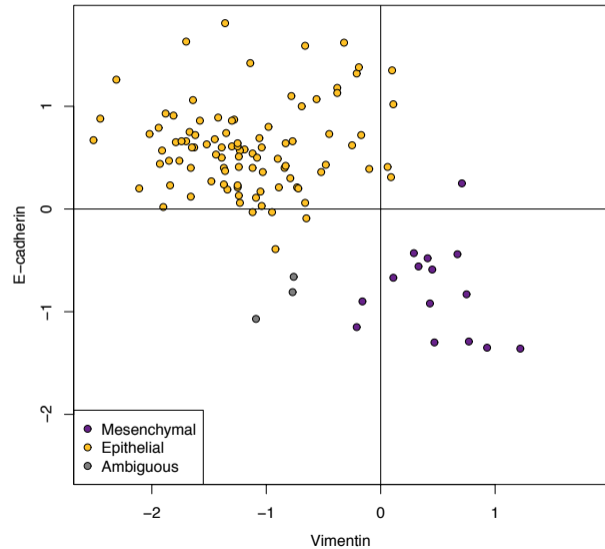

Supplement: Additional file 2 — Tumor type characterization of mouse mammary tumors by Vimentin and E-Cadherin expression analysis. To determine tumor type, log2 ratios of Vimentin and E-Cadherin expression were acquired by hybridization of mRNA of a subset of tumors on Mouse Operon V3 Oligo arrays and were plotted against each other. a) Brca1Δ/Δ;p53Δ/Δ, Brca2Δ/Δ;p53Δ/Δ and p53Δ/Δ mouse tumors were plotted by their E-cadherin and Vimentin expression levels. b) If the difference between E-cadherin and Vimentin expression levels was less than 0.5 on a log scale these tumors were called 'ambiguous'. Tumors were scored 'Mesenchymal' if the Vimentin expression level was >0.5 higher than E-cadherin expression level. Likewise, tumors were scored 'Epithelial' when the E-cadherin expression level was >0.5 higher than the Vimentin expression. [file 1471-2407-10-455-S2.PDF]
