# Supplementary material for: Cross-species comparison of aCGH data from mouse and human BRCA1- and BRCA2-mutated breast cancers
Source: BMC Cancer. 2010 Aug 24;10:455. doi: 10.1186/1471-2407-10-455 (PMC2940799; doi:10.1186/1471-2407-10-455)
Supplement: Additional file 3 — Tumor types of mouse mammary tumors determined by histopathology and gene expression analysis. Mouse mammary tumor types were determined by histopathology and expression data. Histopathology was done as previously described in [10]. Expression data was acquired by hybridization of mRNA of a subset of tumors on Mouse Operon V3 Oligo arrays: If the difference between E-cadherin and Vimentin expression levels was less than 0.5 on a log scale we called these tumors 'ambiguous'. Tumors were scored 'Mesenchymal' if the Vimentin expression level was >0.5 higher than E-cadherin expression level. Likewise, tumors were scored 'Epithelial' when the E-cadherin expression level was >0.5 higher than the Vimentin expression level. The line numbers relate to the line-plots shown in Figure 2, line number 1 on top for each tumor group. [file 1471-2407-10-455-S3.PDF]

Brca1$^{\Delta/\Delta}$;p53$^{\Delta/\Delta}$ (n=35)

Tumor Name	histopathology	VimMinEcad	Vimentin/ Ecadherin expression	Figure Line
03JOS242	Adenomyoepithelioma	-2.48	Epithelial	1
01JOS06	Adenomyoepithelioma	-2.42	Epithelial	2
04LIU77	carcinoma	NA	NA	3
05LIU31	carcinoma	NA	NA	4
00JOS164_2	Carcinoma	-0.87	Epithelial	5
01JOS25	Carcinoma	-1.58	Epithelial	6
01JOS94	Carcinoma	-1.46	Epithelial	7
00JOS164_1	Carcinoma	-1.89	Epithelial	8
01JOS13	Carcinoma	-0.94	Epithelial	9
00JOS94	Carcinoma	-1.66	Epithelial	10
01JOS47	Carcinoma	-0.92	Epithelial	11
01JOS20	Carcinoma	-0.89	Epithelial	12
01JOS08	Carcinoma	-0.35	Epithelial	13
00JOS135	Carcinoma	-1.39	Epithelial	14
01JOS45	Carcinoma	-1.43	Epithelial	15
03JOS272	Carcinoma	-2.72	Epithelial	16
03JOS140	Carcinoma	-2.44	Epithelial	17
04LIU62	Carcinoma	-2.44	Epithelial	18
04JOS22	Carcinoma	-1.52	Epithelial	19
04LIU65	Carcinoma	-1.2	Epithelial	20
04LIU59	Carcinoma	-3.18	Epithelial	21
03JOS174	Carcinoma	-3.33	Epithelial	22
03JOS117	Carcinoma	-3.33	Epithelial	23
04LIU56	Carcinoma	-1.75	Epithelial	24
04JOS09	Carcinoma	-1.56	Epithelial	25
05LIU02	Carcinoma	-1.29	Epithelial	26
04LIU66	Carcinoma	-2.73	Epithelial	27
04LIU61	Carcinoma	-0.91	Epithelial	28
04LIU55	Carcinoma	-1.77	Epithelial	29
04LIU17	Carcinoma	-1.1	Epithelial	30
04LIU73	Carcinoma	-1.39	Epithelial	31
04JOS10	Carcinoma	-2.56	Epithelial	32
03JOS171	Carcinoma	-1.99	Epithelial	33
00JOS88	Carcinosarcoma	-1.94	Epithelial	34
01JOS79	NA	NA	NA	35

Brca2$^{\Delta/\Delta}$;p53$^{\Delta/\Delta}$ (n=62)

Tumor Name	histopathology	VimMinEcad	Vimentin/ Ecadherin expression	Figure Line
99JOS16	carcinoma	NA	NA	1
06HNE06_2	carcinoma	NA	NA	2
06LIU54_1	carcinoma	NA	NA	3
06HNE01	carcinoma	NA	NA	4
06LIU67_2	carcinoma	NA	NA	5
06HNE06_1	carcinoma	NA	NA	6
06HNE02_2	carcinoma	NA	NA	7
06HNE04	carcinoma	NA	NA	8
05HNE22	carcinoma	NA	NA	9
06HNE16	carcinoma	NA	NA	10
06HNE13	carcinoma	NA	NA	11

06EVE18	carcinoma	NA	NA	12
06HNE14	carcinoma	NA	NA	13
06HNE15	carcinoma	NA	NA	14
06HNE05_2	carcinoma	NA	NA	15
06LIU65_2	carcinoma	NA	NA	16
06HNE17	carcinoma	NA	NA	17
06LIU51	carcinoma	NA	NA	18
05LIU56	carcinoma	NA	NA	19
06LIU50	carcinoma	NA	NA	20
06LIU61	carcinoma	NA	NA	21
06HNE11	carcinoma	NA	NA	22
06HNE02_1	carcinoma	NA	NA	23
06HNE05_1	carcinoma	NA	NA	24
03JOS60	carcinoma	-2.15	Epithelial	25
02JOS196_1	carcinoma	-1.53	Epithelial	26
03JOS83	carcinoma	-1.63	Epithelial	27
02JOS196_2	carcinoma	-1.37	Epithelial	28
03JOS63_2	carcinoma	-0.91	Epithelial	29
03JOS63_1	carcinoma	-1.78	Epithelial	30
99JOS45	carcinoma	-2.25	Epithelial	31
00JOS07	carcinoma	-1.88	Epithelial	32
99JOS23_1	carcinoma	-1.77	Epithelial	33
99JOS33	carcinoma	-1.48	Epithelial	34
99JOS54	carcinoma	-1.51	Epithelial	35
99JOS51	carcinoma	-0.1	Ambiguous	36
00JOS08	carcinoma	-0.72	Epithelial	37
99JOS34	carcinoma	-1.65	Epithelial	38
99JOS19	carcinoma	-2.23	Epithelial	39
99JOS30	carcinoma	-1.24	Epithelial	40
99JOS50	carcinoma	-3.17	Epithelial	41
00JOS06	carcinoma	-2.31	Epithelial	42
99JOS17_1	carcinoma	0.94	Mesenchymal	43
00JOS39	carcinoma	-1.91	Epithelial	44
99JOS17_2	carcinoma	-0.56	Epithelial	45
00JOS05	carcinoma	-1.25	Epithelial	46
99JOS32	carcinoma	-0.92	Epithelial	47
00JOS22	carcinoma	-1.09	Epithelial	48
99JOS49_1	carcinoma	-1.47	Epithelial	49
00JOS12	carcinosarcoma	NA	NA	50
06HNE07	carcinosarcoma	NA	NA	51
06HNE03	carcinosarcoma	NA	NA	52
03JOS143	carcinosarcoma	NA	NA	53
99JOS23_2	carcinosarcoma	1.77	Mesenchymal	54
06LIU65_1	NA	NA	NA	55
06LIU55	NA	NA	NA	56
06LIU67_1	NA	NA	NA	57
05LIU62	NA	NA	NA	58
06LIU66	NA	NA	NA	59
03JOS137	NA	-1.78	Epithelial	60
99JOS49_2	NA	-0.88	Epithelial	61
01JOS40	NA	-1.8	Epithelial	62

p53$^{\Delta/\Delta}$ (n=33)				
**Tumor Name**	**histopathology**	**VimMinEcad**	**Vimentin/ Ecadherin expression**	**Figure Line**
01JOS169	Adenomyoepithelioma	-2.7	Epithelial	1
03JOS154_3	Adenomyoepithelioma	-1.57	Epithelial	2
03JOS155_1	Adenomyoepithelioma	-0.49	Epithelial	3
01JOS127_1	Carcinoma	-3.57	Epithelial	4
01JOS165	Carcinoma	-0.02	Ambiguous	5
01JOS181_1	Carcinoma	-2.07	Epithelial	6
01JOS181_2	Carcinoma	-2.32	Epithelial	7
03JOS105	Carcinoma	-1.25	Epithelial	8
03JOS155_2	Carcinoma	-2.25	Epithelial	9
04LIU54	Carcinoma	-1.07	Epithelial	10
06HNE12	Carcinoma	NA	NA	11
03JOS26	Carcinoma	NA	NA	12
05LIU51	carcinoma	NA	NA	13
02JOS23	Carcinoma	NA	NA	14
01JOS65	Carcinosarcoma	1.58	Mesenchymal	15
01JOS66	Carcinosarcoma	0.04	Ambiguous	16
01JOS80_1	Carcinosarcoma	0.89	Mesenchymal	17
01JOS80_2	Carcinosarcoma	2.06	Mesenchymal	18
02JOS92	Carcinosarcoma	0.78	Mesenchymal	19
03JOS03	Carcinosarcoma	0.72	Mesenchymal	20
03JOS14	Carcinosarcoma	1.11	Mesenchymal	21
03JOS29	Carcinosarcoma	1.04	Mesenchymal	22
03JOS154_1	Carcinosarcoma	0.74	Mesenchymal	23
03JOS154_2	Carcinosarcoma	0.89	Mesenchymal	24
05LIU55	carcinosarcoma	NA	NA	25
05LIU50	Carcinosarcoma	NA	NA	26
05LIU25	carcinosarcoma	NA	NA	27
05LIU28	Carcinosarcoma	NA	NA	28
03JOS58	NA	NA	NA	29
05LIU26S	NA	NA	NA	30
05LIU26	NA	NA	NA	31
01JOS127_2	NA	NA	NA	32
03JOS219	NA	NA	NA	33
