# Supplementary material for: Cross-species comparison of aCGH data from mouse and human BRCA1- and BRCA2-mutated breast cancers
Source: BMC Cancer. 2010 Aug 24;10:455. doi: 10.1186/1471-2407-10-455 (PMC2940799; doi:10.1186/1471-2407-10-455)
Supplement: Additional file 4 — KC-SMART analysis of mouse mammary tumors. The significant CNAs of Brca1Δ/Δ;p53Δ/Δ, Brca2Δ/Δ;p53Δ/Δ and p53Δ/Δ mouse tumor groups were obtained by running the KC-SMART algorithm over the BAC data from all tumors in each tumor group using a kernel width of 20 Mb. Significant regions are determined by the intercept of the KSE curve and the significance cutoff calculated for each tumor group, and for gains and losses separately. The upper panel lists recurrent gains and the bottom panel lists the recurrent losses. These regions correspond to the color-matched bars on top (gains) and on the bottom (losses) of the KSE curves of the tumor groups shown in Figure 1d and the red bars of the cross species comparison in Figure 5. [file 1471-2407-10-455-S4.PDF]

region	Brca1$^{\Delta/\Delta}$;p53$^{\Delta/\Delta}$ gains		Brca2$^{\Delta/\Delta}$;p53$^{\Delta/\Delta}$ gains		p53$^{\Delta/\Delta}$ gains	
chromosome	start (Mb)	end (Mb)	start (Mb)	end (Mb)	start (Mb)	end (Mb)
1	11.46	28.71				
	30.86	39.26				
	51.06	97.66	52.26	84.46		
	117.46	138.01				
	152.66	159.46				
2	151.40	181.50	153.45	181.50		
3			27.77	31.82		
5			48.68	54.93		
			62.98	79.78		
	122.83	151.53	99.13	151.53	111.18	151.53
6	20.65	149.40	29.90	38.70	9.45	24.40
			82.75	149.40	110.95	149.40
8			0.00	21.85	0.00	20.65
9	11.17	15.47	6.12	15.77	6.12	13.42
	118.17	123.57			118.07	123.42
10	36.65	69.75	9.00	77.95		
11	102.40	118.40	112.00	118.40	111.35	118.40
12	114.56	117.86				
15	23.31	88.11	46.11	89.56	53.96	101.61
17	26.76	36.11	28.26	33.51		
18					18.25	25.80
19	3.35	7.15	3.35	6.95		
	21.90	31.05				
	35.35	61.00	36.80	61.00		
X	20.26	28.81				

region	Brca1$^{\Delta/\Delta}$;p53$^{\Delta/\Delta}$ losses		Brca2$^{\Delta/\Delta}$;p53$^{\Delta/\Delta}$ losses		p53$^{\Delta/\Delta}$ losses	
chromosome	start (Mb)	end (Mb)	start (Mb)	end (Mb)	start (Mb)	end (Mb)
3	50.37	106.22	76.97	87.72		
	133.52	159.47	141.22	153.97		
4	55.77	63.57			52.37	60.67
	68.02	110.02	83.62	103.37	80.32	108.77
	121.82	152.97	119.67	149.97	117.42	137.72
7					4.22	12.07
			31.77	38.77	23.92	49.02
					67.77	85.77
	134.62	144.27	134.42	144.27	93.07	144.27
8	33.60	55.10	34.90	131.70	26.30	42.55
	59.80	131.70			46.30	50.10
					67.80	131.70
9	59.62	77.52				
	98.22	111.27				
10	78.05	129.20	73.50	129.20	109.90	129.20
11			43.10	51.50		
			56.50	69.05	62.15	69.25
12	12.01	60.26	4.06	117.86	4.06	117.86
	69.01	110.91				
13	98.08	99.83	62.98	76.93		
	108.03	120.23	88.23	118.03		
14	31.45	123.75	37.65	123.75	52.80	82.20
					117.55	120.40
16	24.57	53.47	26.52	51.37		
	75.92	77.57				
	87.12	97.37	88.72	97.37		
17	45.26	56.36				
	74.96	91.71				
18	37.00	90.60	34.55	86.20	36.85	41.70
					53.90	82.65
20	139.11	152.36	143.46	150.21		
