# Supplementary material for: Cross-species comparison of aCGH data from mouse and human BRCA1- and BRCA2-mutated breast cancers
Source: BMC Cancer. 2010 Aug 24;10:455. doi: 10.1186/1471-2407-10-455 (PMC2940799; doi:10.1186/1471-2407-10-455)
Supplement: Additional file 5 — KC-SMART analysis of human breast tumors. Genomic regions found recurrently gained (top) or lost (bottom) by KC-SMART analysis of human BRCA1-related, BRCA2-related, and control breast tumors. The significant CNAs were determined by running the KC-SMART algorithm over the BAC data from all tumors in each tumor group using a kernel width of 20 Mb. Significant regions are determined by the intercept of the KSE curve and the significance cutoff calculated for each tumor group, and for gains and losses separately. The upper panel lists recurrent gains and the bottom panel lists the recurrent losses. [file 1471-2407-10-455-S5.PDF]

region	BRCA1 gains		BRCA2 gains		control gains	
chromosome	start (Mb)	end (Mb)	start (Mb)	end (Mb)	start (Mb)	end (Mb)
1q	143.61	170.51	143.61	185.51	143.61	225.46
	200.36	206.26	190.21	222.61		
	211.16	221.91				
	229.41	242.11			228.46	242.46
3q	144.47	156.47				
	173.57	183.67				
6p	14.94	22.34	16.49	21.99		
8q	61.52	82.37	59.82	139.87	56.07	139.92
	84.37	140.27				
10p	1.21	12.56			1.21	10.56
11q					79.04	79.09
15q			95.86	99.51		
16p					29.81	31.01
17q			48.24	65.59	31.74	37.04
			75.04	78.29	52.49	63.74
20q			50.60	62.10	51.10	53.55

region	BRCA1 losses		BRCA2 losses		control losses	
chromosome	start (Mb)	end (Mb)	start (Mb)	end (Mb)	start (Mb)	end (Mb)
3p						
4p					16.42	18.82
5q	67.36	75.26				
6q			158.64	160.34		
8p	4.23	7.53	5.33	10.08	3.28	12.83
9p			32.09	41.09	34.34	39.94
9q					67.11	68.06
10q			112.21	124.96		
11p			6.58	10.73		
11q			102.34	119.79	103.54	133.94
			121.49	133.94		
13q	48.66	66.86	19.01	70.41	19.01	37.91
			78.91	90.86	48.31	63.81
			98.41	113.76	111.46	113.76
14q	54.07	60.47	54.12	62.47		
	95.87	101.37	90.52	105.22		
15q	0.01	10.46				
	18.56	23.51	13.86	24.91	13.11	26.06
16q					45.07	63.42
					75.02	80.72
17p					5.25	21.80
18q						
19p					18.58	23.53
19q					32.89	33.94
21q			14.63	15.33	14.63	16.18
22q			21.22	28.57	18.67	35.37
			32.77	36.57		
			45.12	49.42	44.27	49.42
Xp	41.74	50.29	41.89	50.14	42.14	50.49
Xq	96.68	112.08	87.28	102.18	91.18	109.23
	130.78	136.58	132.83	137.63	131.43	138.13
					141.18	146.53
