# Supplementary material for: Cross-species comparison of aCGH data from mouse and human BRCA1- and BRCA2-mutated breast cancers
Source: BMC Cancer. 2010 Aug 24;10:455. doi: 10.1186/1471-2407-10-455 (PMC2940799; doi:10.1186/1471-2407-10-455)
Supplement: Additional file 6 — Regions identified by cross-species KC-SMART analysis. Shown are genes within significantly recurrent regions of gain and loss that overlap between (a) human BRCA1-mutated and mouse Brca1Δ/Δ;p53Δ/Δ tumors, (b) human BRCA2-mutated and mouse Brca2Δ/Δ;p53Δ/Δ tumors, and (c) human control tumors and mouse p53Δ/Δ tumors as shown in Figure 5. The syntenic regions in the human and the mouse genome are listed in columns 1 and 2. The "Orthology" column lists the number of mouse (M) and human (H) genes mapping within each region of overlap, and the number of unique orthologous pairs (pair). Strand inversion between the two species is indicated by 1 (no inversion) or -1 (inversion). Listed are cancer-related genes included in the Atlas of Genetics and Cytogenetics in Oncology and Haematology [44,45], and the Cancer Gene Census (CGC)[47,46]. Annotated cancer genes and CGC genes are shown in bold type, putative cancer genes are shown in normal type. Cancer-related genes that map closest to the human KSE peak are shown in blue and genes that map closest to the mouse KSE peak are shown in red. Genes that map to the mouse AND human KSE peaks are shown in green. Single genes that were not listed in the Atlas or the CGC are shown in italics. [file 1471-2407-10-455-S6.PDF]

a

BRCA1 gains

Human Chr.	Human Start (Mb)	Human End (Mb)	Mouse Chr.	Mouse Start (Mb)	Mouse End (Mb)	Ortholog H	Ortholog M	Ortholog pair	strand	**Annotated / cancer gene census**/Putative Oncogenes in Region of overlap peak location of KSE curves: blue: human, red: mouse, green: both mouse and human
1	148.53	148.55	6	122.87	122.87	1	1	1		*MRPS21*
	154.54	154.54	6	113.59	113.60	1	1	1		VHLL
	200.37	205.60	1	132.22	136.97	70	71	71	-1	PTPN7, UBE2T, PPP1R12B, JARID1B, RABIF, ADIPOR1, PPFIA4, MYOG, CHI3L1, CHIT1, BTG2, FMOD, OPTC, REN, PLEKHA6, PPP1R15B, PIK3C2B, MDM4, LRRN2, CNTN2, RBBP5, RIPK5, NUAK2, LEMD1, PCTK3, ELK4, **SLC45A3**, RAB7L1, CTSE, SRGAP2, IKBKE, RASSF5, DYRK3, MAPKAPK2, IL10, IL24, FAIM3, PIGR, C4BPA, CD55
8	70.54	76.11	1	12.68	17.75	21	21	21	1	SULF1, PRDM14, **NCOA2**, TRAM1, TERF1, RDH10, TCEB1
	97.58	139.58	15	32.87	71.56	121	123	123	1	SDC2, TSPYL5, MTDH, LAPTM4B, HRSP12, **COX6C**, FBXO43, SPAG1, RNF19A, PABPC1, YWHAZ, RRM2B, UBR5, KLF10, ATP6V1C1, **BAALC**, FZD6, **CTHRC1**, WDSOF1, RIMS2, DPYS, LRP12, ABRA, ANGPT1, RSPO2, EIF3E, TMEM74, NUDCD1, **EBAG9**, TRPS1, EIF3H, RAD21, **EXT1**, **TNFRSF11B**, MAL2, NOV, **ENPP2**, COL14A1, MTBP, HAS2, ZHX2, DERL1, FAM83A, ATAD2, FBXO32, ANXA13, TRMT12, **RNF139**, MTSS1, SQLE, KIAA0196, NSMCE2, TRIB1, FAM84B, **MYC**, GSDMC, ASAP1, ADCY8, KCNQ3, TG, WISP1, NDRG1, KHDRBS3
10	5.00	5.25	1	64.95	64.97	4	1	4		AKR1C1, AKR1C2, **AKR1C3**

BRCA1 losses

Human Chr.	Human Start (Mb)	Human End (Mb)	Mouse Chr.	Mouse Start (Mb)	Mouse End (Mb)	Ortholog H	Ortholog M	Ortholog pair	strand	**Annotated / cancer gene census**/Putative Oncogenes in Region of overlap peak location of KSE curves: blue: human, red: mouse, green: both mouse and human
5	70.71	70.71	10	87.52	87.52	1	1	1		*PMCHL2*
	72.45	74.20	13	98.17	99.79	9	9	9	-1	BTF3, **ENC1**
13	48.72	51.23	14	58.51	61.91	17	18	18	1	PHF11, RCBTB1, ARL11, KPNA3, TRIM13, KCNRG, RNASEH2B, **INTS6**
	52.12	52.52	14	78.32	78.76	4	4	4	1	PCDH8, OLFM4
	57.10	66.70	14	83.25	92.77	5	5	5	1	*PCDH17, DIAPH3, TDRD3, PCDH20, PCDH9*
14	54.10	57.69	14	45.80	48.70	19	20	20	1	GCH1, MAPK1IP1L, LGALS3, DLGAP5, FBXO34, **KTN1**, PELI2, OTX2
	54.59	54.61	7	138.67	138.68	1	1	1		MAPK1IP1L
	57.74	60.26	12	71.86	74.03	21	21	21	1	ARID4A, TIMM9, DACT1, RTN1, PPM1A, **SIX1**
	95.90	101.10	12	106.09	110.73	20	20	20	1	AK7, **VRK1**, **BCL11B**, CCNK, EML1, EVL, YY1, C14orf68, DLK1, DIO3
15	19.80	19.87	14	49.20	49.24	2	3	3	1	*OR4N4, OR4M2*
X	47.54	47.55	4	132.40	132.47	1	1	1		*WASF4*
	100.41	100.43	10	115.52	115.52	1	1	1		*TAF7L*
	102.36	102.36	16	32.11	32.11	1	1	1		*BEX4*
	104.54	104.54	14	66.67	66.70	1	1	1		KCTD9L
	106.40	106.40	4	122.50	122.50	1	1	1		*MYCL2*
	110.81	111.81	X	139.56	140.60	5	5	5	1	*CXorf45, AC005191.1, ALG13, TRPC5, ZCCHC16, LHFPL1*
	135.78	135.79	8	81.40	81.41	1	1	1		RBMX
	135.78	135.79	14	42.59	42.60	1	1	1		RBMX

b

BRCA2 gains

Human Chr.	Human Start (Mb)	Human End (Mb)	Mouse Chr.	Mouse Start (Mb)	Mouse End (Mb)	Ortholog H	Ortholog M	Ortholog pair	strand	**Annotated / cancer gene census**/Putative Oncogenes in Region of overlap peak location of KSE curves: blue: human, red: mouse, green: both mouse and human
1	148.53	148.55	6	122.87	122.87	1	1	1		*MRPS21*
	154.54	154.54	6	113.59	113.60	1	1	1		VHLL
	158.44	158.45	5	78.59	78.59	1	1	1		PEA15
	220.91	220.95	8	4.09	4.09	1	1	1		*AIDA*
8	113.30	139.58	15	47.41	71.56	65	67	67	1	TRPS1, EIF3H, RAD21, **EXT1**, **TNFRSF11B**, MAL2, NOV, **ENPP2**, COL14A1, MTBP, HAS2, ZHX2, DERL1, FAM83A, ATAD2, FBXO32, ANXA13, TRMT12, **RNF139**, MTSS1, SQLE, KIAA0196, NSMCE2, TRIB1, FAM84B, **MYC**, GSDMC, ASAP1, ADCY8, KCNQ3, TG, WISP1, NDRG1, KHDRBS3
20	51.02	57.33	2	169.32	174.43	39	38	39	1	**ZNF217**, BCAS1, CYP24A1, **AURKA**, CSTF1, TFAP2C, BMP7, CTCFL, PMEPA1, C20orf85, RAB22A, GNAS, CTSZ, TUBB1, EDN3
	57.59	62.04	2	178.07	181.48	51	51	51	1	PPP1R3D, CDH4, PSMA7, **SS18L1**, HRH3, ADRM1, LAMA5, GATA5, NTSR1, OGFR, DIDO1, BIRC7, CHRNA4, **EEF1A2**, PTK6, SRMS, RTEL1, ARFRP1, TPD52L2, DNAJC5

BRCA2 losses

Human Chr.	Human Start (Mb)	Human End (Mb)	Mouse Chr.	Mouse Start (Mb)	Mouse End (Mb)	Ortholog H	Ortholog M	Ortholog pair	strand	**Annotated / cancer gene census**/Putative Oncogenes in Region of overlap peak location of KSE curves: blue: human, red: mouse, green: both mouse and human
8	7.89	7.89	14	61.93	61.94	1	1	1		*AC130365.5-202*
	8.21	9.68	8	36.30	37.62	5	5	5	-1	MFHAS1, PPP1R3B, TNKS
9	39.06	39.28	13	64.79	64.92	1	1	1		*CNTNAP3*
11	6.59	6.59	10	82.38	82.38	1	1	1		TAF10
	111.56	111.56	14	66.67	66.70	1	1	1		*AP002884.5*
	117.78	117.79	10	95.57	95.57	1	1	1		*ATP5L*
13	19.15	21.17	14	55.68	57.07	17	17	17	1	ZMYM2, GJB2, GJB6, IFT88, (SAP18), LATS2, FGF9
	22.65	25.52	14	58.58	60.21	16	15	16	-1	SACS, TNFRSF19, MIPEP, SPATA13, C1QTNF9, PARP4, ATP12A, RNF17, CENPJ, ATP8A2
	34.41	39.22	3	53.07	56.27	22	22	22	-1	NBEA, DCLK1, SPG20, CCNA1, SMAD9, FAM48A, POSTN, TRPC4, **LHFP**
	36.58	36.58	18	61.68	61.71	1	1	1		*CSNK1A1L*
	40.40	48.68	14	71.27	78.32	44	45	45	-1	ELF1, C13orf15, AKAP11, TNFSF11, EPSTI1, DNAJC15, TSC22D1, SPERT, CPB2, **LCP1**, HTR2A, ITM2B, **RB1**, P2RY5, RCBTB2
	45.82	45.91	16	31.17	31.20	1	1	1		*AL139801.17*
	45.96	45.96	16	38.27	38.27	1	1	1		*RP11-189B4.3*
	48.72	51.23	14	58.51	61.91	17	18	18	1	PHF11, RCBTB1, ARL11, KPNA3, TRIM13, KCNRG, RNASEH2B, **INTS6**
	52.12	69.58	14	78.32	95.40	10	10	10	1	PCDH8, OLFM4, KLHL1
	78.95	87.13	14	104.14	110.56	5	5	5		SPRY2
	98.70	101.17	14	121.05	123.11	12	12	12	1	EBVS1, PCCA
14	54.38	57.69	14	46.08	48.70	18	19	19	1	GCH1, MAPK1IP1L, LGALS3, DLGAP5, FBXO34, **KTN1**, PELI2, OTX2
	54.59	54.61	7	138.67	138.68	1	1	1		MAPK1IP1L
	57.74	61.67	12	71.86	75.21	30	30	30	1	ARID4A, TIMM9, DACT1, RTN1, PPM1A, **SIX1**, MNAT1, PRKCH, HIF1A
	90.65	105.07	12	101.18	113.64	112	122	122	1	GPR68, FBLN5, **TRIP11**, ATXN3, RIN3, LGMN, GOLGA5, CHGA, MOAP1, BTBD7, ASB2, OTUB2, DDX24, IFI27, SERPINA1, SERPINA9, SERPINA5, SERPINA3, GSC, DICER1, (TCL1B), **(TCL1A)**, BDKRB2, BDKRB1, AK7, **VRK1**, **BCL11B**, CCNK, EML1, EVL, YY1, C14orf68, DLK1, DIO3, PPP2R5C, **HSP90AA1**, RAGE, **TRAF3**, CDC42BPB, TNFAIP2, MARK3, CKB, C14orf172, BAG5, KLC1, **XRCC3**, PPP1R13B, SIVA1, **AKT1**, GPR132, **JAG2**, BRF1, **MTA1**
15	18.43	18.43	12	115.12	115.83	1	2	2		*VSIG7*
	19.80	19.87	14	49.20	49.24	2	3	3	1	*OR4N4, OR4M2*
22	21.30	21.99	10	74.40	75.03	6	5	6	mix	RTDR1, GNAZ, RAB36, **BCR**
	22.03	22.05	8	96.37	96.42	1	2	2		*AP000344.1-203*
	22.42	22.97	10	75.03	75.39	17	15	17	-1	VPREB3, **MMP11**, **SMARCB1**, MIF, DDT, GSTT2, GSTT1
	23.00	23.35	10	74.67	75.03	7	7	7	1	ADORA2A, GGT1
	33.79	34.28	8	77.77	78.12	6	6	6		LARGE, HMGXB4, TOM1, HMOX1, MCM5, RASD2
X	47.54	47.55	4	132.40	132.47	1	1	1		*WASF4*
	100.41	100.43	10	115.52	115.52	1	1	1		*TAF7L*
	135.78	135.79	8	81.40	81.41	1	1	1		RBMX
	135.78	135.79	14	42.59	42.60	1	1	1		RBMX

c

human control and mouse p53$^{\Delta/\Delta}$ gains

Human Chr.	Human Start (Mb)	Human End (Mb)	Mouse Chr.	Mouse Start (Mb)	Mouse End (Mb)	Ortholog H	Ortholog M	Ortholog pair	strand	**Annotated / cancer gene census**/Putative Oncogenes in Region of overlap peak location of KSE curves: blue: human, red: mouse, green: both mouse and human
1	148.53	148.55	6	122.87	122.87	1	1	1		*MRPS21*
	154.54	154.54	6	113.59	113.60	1	1	1		VHLL
	220.91	220.95	8	4.09	4.09	1	1	1		*AIDA*
8	120.00	139.58	15	54.08	71.56	55	57	57	1	**TNFRSF11B**, MAL2, NOV, **ENPP2**, COL14A1, MTBP, HAS2, ZHX2, DERL1, FAM83A, ATAD2, FBXO32, ANXA13, TRMT12, **RNF139**, MTSS1, SQLE, KIAA0196, NSCME2, TRIB1, FAM84B, **MYC**, GSDMC, ASAP1, ADCY8, KCNQ3, TG, WISP1, NDRG1, KHDRBS3

human control and mouse p53$^{\Delta/\Delta}$ losses

Human Chr.	Human Start (Mb)	Human End (Mb)	Mouse Chr.	Mouse Start (Mb)	Mouse End (Mb)	Ortholog H	Ortholog M	Ortholog pair	strand	**Annotated / cancer gene census**/Putative Oncogenes in Region of overlap peak location of KSE curves: blue: human, red: mouse, green: both mouse and human
8	7.18	7.87	7	103.12	104.73	3	5	15	mix	*AF228730.6, AC130360.4-202, AC130365.5-201*
	7.89	7.89	14	61.93	61.94	1	1	1		*AC130365.5-202*
	8.21	9.68	8	36.30	37.62	5	5	5	-1	MFHAS1, PPP1R3B, TNKS
	9.95	11.97	14	61.95	63.41	18	18	18	-1	MSRA, SOX7, PINX1, MTMR9, BLK, GATA4, NEIL2, FDFT1, **CTSB**
	12.03	12.03	7	103.12	104.73	1	5	5		*USP17*
	12.21	12.22	14	61.95	61.96	1	1	1		*DEFB130*
	12.62	12.66	8	37.69	37.70	1	1	1		*LONRF1*
11	111.56	111.56	14	66.67	66.70	1	1	1		*AP002884.5*
13	19.15	25.52	14	55.68	58.59	33	32	33	1	ZMYM2, GJB2, GJB6, IFT88, LATS2, FGF9, SACS, TNFRSF19, MIPEP, SPATA13, C1QTNF9, PARP4, ATP12A, RNF17, CENPJ, ATP8A2
	36.58	36.58	18	61.68	61.71	1	1	1		*CSNK1A1L*
	48.45	48.68	14	71.27	71.45	1	1	1		*FNDC3A*
	48.72	51.23	14	58.51	61.91	17	18	18	1	PHF11, RCBTB1, ARL11, KPNA3, TRIM13, KCNRG, RNASEH2B, **INTS6**
	52.12	52.52	14	78.32	78.76	4	4	4	1	PCDH8, OLFM4
15	18.43	18.43	12	115.12	115.83	1	2	2		VSIG7
	20.74	20.76	7	81.44	81.47	1	1	1		*RP11-365K22.1, WHAMML1*
16	45.25	60.63	8	88.15	102.31	88	94	95	1	ORC6L, DNAJA2, SIAH1, ADCY7, BRD7, NOD2, **CYLD**, **RBL2**, IRX5, **MMP2**, SLC6A2, CES1, GNAO1, AMFR, MT3, NUP93, SLC12A3, CCL22, CX3CL1, CCL17, CIAPIN1, GPR56, KATNB1, MMP15, CSNK2A2, NDRG4, CDH8
	75.80	80.69	8	116.52	120.65	21	21	21	1	ADAMTS18, **WWOX**, **MAF**, DYNLRB2, ATMIN, PLCG2, HSD17B2
17	7.68	18.65	11	62.70	69.23	71	71	72	-1	JMJD3, CHD3, GUCY2D, ALOX15B, HES7, **PER1**, AURKB, ARHGEF15, MYH10, PIK3R5, NTN1, WDR16, RCVRN, **GAS7**, MYH2, SCO1, **MAP2K4**, **ELAC2**, COX10, PMP22, ADORA2B, ZSWIM7, NCOR1, PIGL, UBB, TRPV2, MPRIP, **FLCN**, COPS3, RASD1, PEMT, RAI1, SREBF1, ALKBH5, TOP3A, SHMT1, KRT17
	10.65	10.65	7	4.39	4.39	1	1	1		*AC015908.10*
19	18.58	19.64	8	72.72	73.42	29	29	29	-1	**CRTC1**, COMP, UPF1, GDF1, COPE, DDX49, SLC25A42, MEF2B, RFXANK, PBX4, LPAR2
22	22.03	22.05	8	96.40	96.38	1	2	2		*AP000344.1-203*
	32.00	34.28	8	75.71	78.12	7	7	7	1	LARGE, HMGXB4,TOM1, HMOX1, MCM5, RASD2
X	47.54	47.55	4	132.40	132.47	1	1	1		*WASF4*
	100.41	100.43	10	115.52	115.52	1	1	1		*TAF7L*
	104.54	104.54	14	66.67	66.70	1	1	1		KCTD9L
	106.40	106.40	4	122.50	122.50	1	1	1		*MYCL2*
	135.78	135.79	8	81.40	81.41	1	1	1		RBMX
