# Supplementary material for: Cross-species comparison of aCGH data from mouse and human BRCA1- and BRCA2-mutated breast cancers
Source: BMC Cancer. 2010 Aug 24;10:455. doi: 10.1186/1471-2407-10-455 (PMC2940799; doi:10.1186/1471-2407-10-455)
Supplement: Additional file 7 — Comparative-KC-SMART analysis of mouse mammary tumors. The significant differential gains and losses of Brca1Δ/Δ;p53Δ/Δ vs. p53Δ/Δ and Brca2Δ/Δ;p53Δ/Δ vs. p53Δ/Δ mouse tumor groups were obtained by running the comparative-KC-SMART algorithm over the BAC data from all tumors in each tumor group using a kernel width of 20 Mb. The upper panel shows recurrent differential gains and the bottom panel shows the differential losses. These regions correspond to the red bars plotted on top (gains) and on the bottom (losses) of the corresponding mouse KSE curves used in the cross species comparison in Figure 6. [file 1471-2407-10-455-S7.PDF]

region	Brca1$^{\Delta/\Delta}$;p53$^{\Delta/\Delta}$ gains		Brca2$^{\Delta/\Delta}$;p53$^{\Delta/\Delta}$ gains	
chromosome	start (Mb)	end (Mb)	start (Mb)	end (Mb)
1	3.51<br>119.51<br>151.11<br>186.56	99.21<br>140.31<br>166.66<br>192.96	50.11	86.96
2	150.80	181.50	153.35	181.50
5	135.13	151.53	137.73	151.53
6	27.05<br>64.50<br>92.15<br>110.55<br>129.05	60.30<br>70.55<br>104.00<br>121.80<br>149.40	91.00	99.95
10			17.45	74.80
11	116.85	118.40		
15	51.16	54.16		

region	Brca1$^{\Delta/\Delta}$;p53$^{\Delta/\Delta}$ losses		Brca2$^{\Delta/\Delta}$;p53$^{\Delta/\Delta}$ losses	
chromosome	start (Mb)	end (Mb)	start (Mb)	end (Mb)
2	87.30	95.20		
3	37.32<br>133.37	93.52<br>159.47		
4	73.22	107.27		
8	85.95<br>108.00	100.40<br>129.10	79.65	131.70
9	59.17	90.82		
10	82.10	129.20	80.55	129.20
13	111.23	120.23	89.38	103.38
14	37.60	123.75	54.15<br>93.95	90.95<br>123.75
16	27.62	51.82		
17	48.46<br>81.41	63.76<br>94.36		
18	51.10	90.60		
20	89.91<br>124.31	106.66<br>165.31		
