# Supplementary material for: Cross-species comparison of aCGH data from mouse and human BRCA1- and BRCA2-mutated breast cancers
Source: BMC Cancer. 2010 Aug 24;10:455. doi: 10.1186/1471-2407-10-455 (PMC2940799; doi:10.1186/1471-2407-10-455)
Supplement: Additional file 8 — Comparative-KC-SMART analysis of human breast tumors. Comparative KC-SMART analysis of human BRCA1-related and BRCA2 related breast tumors compared with control breast tumors. Regions of differential aberrations between BRCA1 and control breast tumors, between BRCA2 and control breast tumors. Top panel: Differential gains. Bottom panel: Differential losses. [file 1471-2407-10-455-S8.PDF]

region	BRCA1 gains		BRCA2 gains	
chromosome	start (Mb)	end (Mb)	start (Mb)	end (Mb)
1p	42.06	78.61		
	87.31	101.91		
1q	166.56	168.31	167.21	168.81
2p	54.31	64.36		
3q	131.87	164.22		
	168.37	190.17		
5q			151.31	158.31
6p	1.24	31.14		
	37.04	45.29		
	53.34	58.59		
6q	79.29	79.99		
	83.69	86.39		
	119.49	129.64		
	139.84	144.74		
7q	100.48	107.68		
	112.38	142.53		
	149.88	157.53		
8q	100.07	137.42		
9p			2.49	4.44
10p	1.21	7.81		
	24.51	32.31		
11p	25.63	33.63		
12p	0.15	19.10		
13q	86.26	86.66		
	96.46	105.21		
15q			94.36	99.51
16q	87.22	88.42		
18q	75.18	75.58		
19p	13.18	21.63		
19q	32.89	44.54	34.19	39.79
21q	17.88	28.38		
	35.13	38.43		

region	BRCA1 losses		BRCA2 losses	
chromosome	start (Mb)	end (Mb)	start (Mb)	end (Mb)
4q	103.70	108.00		
	115.75	129.85		
	158.85	167.15		
	171.10	179.00		
5p	34.47	42.32		
5q	49.91	148.86		
	160.61	178.21		
7p	3.46	25.71		
11p	51.13	51.28		
12q	53.05	61.90		
	86.30	87.70		
	94.85	103.10		
13q			28.61	62.26
14q	37.87	42.87		
	48.72	106.37	52.67	105.22
15p	0.00	11.11		
15q	33.61	51.16		
20p	0.33	6.48	0.33	4.13
	18.43	24.93		
20q	35.00	40.55		
Xp	53.79	66.43		
Xq	68.53	74.13		
	85.43	85.88		
	112.68	115.83		
