# Supplementary material for: Cross-species comparison of aCGH data from mouse and human BRCA1- and BRCA2-mutated breast cancers
Source: BMC Cancer. 2010 Aug 24;10:455. doi: 10.1186/1471-2407-10-455 (PMC2940799; doi:10.1186/1471-2407-10-455)
Supplement: Additional file 9 — Regions identified by cross-species comparative-KC-SMART analysis. Shown are (a) genes that map to differentially recurrent CNAs found in both mouse Brca1Δ/Δ;p53Δ/Δ vs. p53Δ/Δ mammary tumors and human BRCA1-mutated vs. control breast tumors and (b) genes mapping to differentially recurrent CNAs found in both mouse Brca2Δ/Δ;p53Δ/Δ vs. p53Δ/Δ mammary tumors and BRCA2-mutated vs. control breast tumors, shown in Figure 7 [file 1471-2407-10-455-S9.PDF]

a

BRCA1 gains

Human			Mouse			Orthology				**Annotated**/Putative Oncogenes in Region
Chr.	Start (Mb)	End (Mb)	Chr.	Start (Mb)	End (Mb)	H	M	pair	strand	peak location of KSE curves: blue: human, red: mouse, green: both mouse and human
1	45.01	45.02	2	175.02	175.03	1	1	1		RPS8
	58.81	58.82	6	67.46	67.47	1	1	1		TACSTD2
	67.40	68.07	6	66.95	67.42	5	5	5	-1	IL23R, IL12RB2, SERBP1, GADD45A, GNG12
	166.93	168.31	1	165.62	166.66	14	14	14	-1	ATP1B1, BLZF1, F5, SELP, SELL, **SELE**
6	56.43	57.62	1	33.40	34.25	6	6	6	-1	DST, ZNF451, BAG2, RAB23, PRIM2
7	100.52	102.09	5	136.37	143.49	25	21	28	-1	SERPINE1, AP1S1, VGF, RABL5, ALKBH4, AC105052.3-2
	125.87	142.43	6	27.23	41.70	107	109	109		ARF5, PAX4, SND1, LRRC4, LEP, IMPDH1, CCDC136, ATP6V1F, IRF5, TNPO3, **SMO,** AHCYL2, NRF1, UBE2H, ZC3HC1, MEST, COPG2, MKLN1, PODXL, EXOC4, AKR1B1, AKR1B10, BPGM, CALD1, NUP205, PTN, DGKI, **CREB3L2, TRIM24**, **KIAA1549,** HIPK2, TBXAS1, RAB19, ADCK2, **BRAF**, TAS2R38, EPHB6, TRPV6
	149.90	150.19	6	48.61	48.86	7	11	11	1	GIMAP4, TMEM176A
8	117.73	120.03	15	51.62	54.11	9	9	9	1	EIF3H, RAD21, **EXT1, TNFRSF11B**
10	5.00	5.25	1	64.95	64.97	4	1	4		AKRCR1, AKR1C2, **AKR1C3**
	27.32	27.43	6	118.47	118.53	1	1	1		*ANKRD26*
11	27.56	27.56	5	138.22	138.22	1	1	1		*IMMP1L*
12	0.17	2.68	6	118.56	121.33	16	16	16	-1	**JARID1A**, B4GALNT3, RAD52, **ERC1,** WNT5B, ADIPOR2
	9.10	9.16	6	121.60	121.64	1	1	1		*AC006057.5-1*
	9.80	18.78	6	129.23	140.01	66	90	103	1	CD69, CLECK12A, CLEC1B, CLEC7A, **AC022075.29, KLRK1,** KLRC4, KLRC3, KLRC2, KLRC1, STYK1, CSDA, **ETV6,** BCL2L14, LRP6, LOH12CR1, CREBL2, GPR19, **CDKN1B**, DDX47, GPRC5A, EMP1, GRIN2B, GUCY2C, ARHGDIB, RERG, PTPRO, EPS8, STRAP, MGST1, LMO3, PLCZ1
13	102.05	102.35	1	43.88	44.16	7	7	7	1	**ERCC5**

BRCA1 losses

Human			Mouse			Orthology				**Annotated**/Putative Oncogenes in Region
Chr.	Start (Mb)	End (Mb)	Chr.	Start (Mb)	End (Mb)	H	M	pair	strand	peak location of KSE curves: blue: human, red: mouse, green: both mouse and human
4	103.77	105.64	3	134.17	135.51	7	7	7	-1	MANBA, UBE2D3, NHEDC2, BDH2, CENPE, CXXC4
	123.75	129.43	3	37.41	41.18	15	16	16	1	FGF2, NUDT6, FAT4, HSPA4L, PLK4
	159.27	163.30	3	76.16	80.03	9	9	9	-1	PPID, RAPGEF2
	165.34	165.34	9	62.17	62.18	1	1	1		ANP32C
5	50.00	58.18	13	111.18	118.14	31	31	31	-1	ITGA1, ITGA2, FST, HSPB3, ESM1, CCNO, DHX29, PPAP2A, DDX4, IL31RA, **IL6ST,** MAP3K1, PLK2, RAB3C
	70.71	70.71	10	87.52	87.52	1	1	1		*PMCHL2*
	75.50	75.51	10	117.22	117.25	1	1	1		*AC113404.3*
	78.44	78.46	18	87.89	87.89	1	1	1		*BHMT*
	102.91	108.55	17	58.68	63.82	4	4	4	1	EFNA5, FER
	108.70	108.77	17	93.07	93.12	1	1	1		*PJA2*
	112.26	112.26	X	159.28	159.30	1	1	1		*ZRSR1*
	119.83	129.55	18	51.24	59.54	30	30	30	1	LOX, CSNK1G3, ZNF608, ALDH7A1, SLC12A2, ADAMTS19
	147.63	148.74	18	61.81	62.74	11	11	11	-1	**SPINK7**, FBXO38, ADRB2
11	51.27	51.27	2	89.21	89.59	1	15	15		*OR4A5*
12	53.96	61.83	10.00	121.85	129.14	100	113	113	-1	ITGA7, BLOC1S1, CD63, GDF11, **SARNP,** DNAJC14, MMP19, DGKA, SILV, CDK2, RAB5B, ERBB3, PA2G4, SMARCC2, RNF41, OBFC2B, CS, IL23A, STAT2, TIMELESS, GLS2, PTGES3, RDH16, ZBTB39, MYO1A, STAT6, LRP1, INHBC, **GLI1**, ARHGAP9, **DDIT3**, PIP4K2C, GEFT, B4GALNT1, OS9, **AGAP2**, TSPAN31, **CDK4**, CYP27B1, FAM119B, CTDSP2, XRCC6BP1, LRIG3, SLC16A7, PPM1H
	54.84	54.84	14	54.37	54.37	1	1	1		*A2M*
	54.95	54.98	10	99.19	99.19	1	1	1		CS
	86.90	87.50	10	99.45	100.05	5	5	5	-1	**KITLG**
	94.86	103.06	10	82.18	92.97	39	39	39	-1	LTA4H, ELK3, PCTK2, TMPO, IKIP, **APAF1**, SCYL2, NR1H4, SLC5A8, UTP20, ARL1, SPIC, CHPT1, **DRAM, AC084398.25-1**, IGF1, **ASCL1**, NT5DC3, TDG, HSP90B1, NFYB
14	51.80	57.69	14	43.77	48.70	34	34	34	1	PTGER2, ERO1L, BMP4, CDKN3, CGRRF1, GCH1, MAPK1IP1L, LGALS3, DLGAP5, FBXO34, **KTN1,** PELI2, OTX2
15	49.76	50.87	9	74.65	75.47	13	13	13	-1	TMOD2, TMOD3, **MAPK6**, BCL2L10, GNB5, ONECUT1
X	53.98	56.61	X	146.86	148.84	16	16	16	1	FGD1, MAGED2, APEX2, MAGEH1, FOXR2, KLF8
	57.63	65.78	X	90.98	93.58	13	12	13	1	ARHGEF9, **FAM123B,** ZC4H2, KIAA1166, **MSN**, EDA2R
	68.64	74.06	X	96.02	100.40	41	41	41	1	EDA, ARR3, KIF4A, DLG3, **FOXO4**, IL2RG, GJB1, **NONO**, TAF1, OGT, **CXCR3,** (ERCC6L), CITED1, HDAC8, CHIC1, RNF12
	71.18	71.18	8	110.33	110.33	1	1	1		*RSP26P11*
	113.72	114.37	X	142.21	142.76	3	3	3	1	IL13RA2

b

BRCA2 losses

Human			Mouse			Orthology				**Annotated**/Putative Oncogenes in Region
Chr.	Start (Mb)	End (Mb)	Chr.	Start (Mb)	End (Mb)	H	M	pair	strand	peak location of KSE curves: blue: human, red: mouse, green: both mouse and human
13	40.40	48.68	14	71.27	78.32	42	43	42	-1	ELF1, C13orf15, AKAP11, TNFSF11, EPSTI1, DNAJC15, TSC22D1, SPERT, CPB2, **LCP1**, HTR2A, ITM2B, **RB1**, P2RY5, RCBTB2
	48.72	51.23	14	58.51	61.91	17	18	18	1	PHF11, RCBTB1, ARL11, KPNA3, TRIM13, KCNRG, RNASEH2B, **INTS6**
	52.12	52.52	14	78.32	78.76	4	4	4	1	PCDH8, OLFM4
	57.10	60.90	14	83.25	87.32	4	4	4	1	
